# Nuclear envelope assembly relies on CHMP-7 in the absence of BAF–LEM-mediated hole closure

**DOI:** 10.1242/jcs.261385

**Published:** 2023-11-13

**Authors:** Sarah R. Barger, Lauren Penfield, Shirin Bahmanyar

**Affiliations:** Yale University, Department of Molecular, Cellular, Developmental Biology, 266 Whitney Ave., New Haven, CT 06511, USA

**Keywords:** BAF, ESCRT, LEM domain, Nuclear envelope

## Abstract

Barrier-to-autointegration factor (BAF) protein is a DNA-binding protein that crosslinks chromatin to allow mitotic nuclear envelope (NE) assembly. The LAP2-emerin-MAN1 (LEM)-domain protein LEMD2 and ESCRT-II/III hybrid protein CHMP7 close NE holes surrounding spindle microtubules (MTs). BAF binds LEM-domain family proteins to repair NE ruptures in interphase, but whether BAF–LEM binding participates in NE hole closure around spindle MTs is not known. Here, we took advantage of the stereotypical event of NE formation in fertilized *Caenorhabditis elegans* oocytes to show that BAF–LEM binding and LEM-2–CHMP-7 have distinct roles in NE closure around spindle MTs. LEM-2 and EMR-1 (homologs of LEMD2 and emerin) function redundantly with BAF-1 (the *C. elegans* BAF protein) in NE closure. Compromising BAF–LEM binding revealed an additional role for EMR-1 in the maintenance of the NE permeability barrier. In the absence of BAF–LEM binding, LEM-2–CHMP-7 was required for NE assembly and embryo survival. The winged helix domain of LEM-2 recruits CHMP-7 to the NE in *C. elegans* and a LEM-2-independent nucleoplasmic pool of CHMP-7 also contributes to NE stability. Thus, NE hole closure surrounding spindle MTs requires redundant mechanisms that safeguard against failure in NE assembly to support embryogenesis.

## Teaser

**Highlighted Article:** In *C. elegans*, nuclear closure around spindle microtubules requires BAF-1 binding to LEM-domain proteins and reveals redundant and unique functions for EMR-1, LEM-2 and CHMP-7 in nuclear maintenance.

## INTRODUCTION

The nuclear envelope (NE) is a domain of the endoplasmic reticulum (ER) that serves as a mechanically stable, semi-permeable barrier to the genome (Ungricht and Kutay, 2017). The outer and inner nuclear membranes of the NE encase a lumen that is shared with the ER. The inner nuclear membrane (INM) has a unique protein composition and is associated with a meshwork of filamentous nuclear lamins. Each cell division in metazoans, the NE and nuclear lamins disassemble to release mitotic chromosomes for capture by spindle microtubules (MTs). After chromosome segregation, the NE forms from ER-derived membranes. Assembly of nuclear pore complexes (NPCs) and closure of NE holes establish the NE permeability barrier. Failure in the barrier function of the NE can lead to DNA damage and disrupt genome regulation, highlighting the importance of understanding mechanisms that seal the NE after mitosis (Gauthier and Comaills, 2021; Rodriguez-Muñoz et al., 2022).

NE formation relies on the double-stranded DNA-binding protein barrier-to-autointegration factor (BAF) protein, which dimerizes to crosslink DNA and binds to a subset of integral INM proteins and lamins (Sears and Roux, 2020). Immediately after exit from mitosis, dephosphorylation of BAF promotes its high-affinity association with segregated chromosome masses (Ahn et al., 2019; Asencio et al., 2012; Marcelot et al., 2021; Snyers et al., 2018). BAF dimers bridge DNA segments to ‘glue’ individual chromosomes together (Samwer et al., 2017). Integral membrane LAP2-emerin-MAN1 (LEM)-domain proteins bind to a groove at the BAF dimer junction through a conserved 40-amino-acid LEM domain. This interaction serves to tether associated ER membranes around the chromatin surface (Barton et al., 2015; Cai et al., 2007; Lin et al., 2000). Nascent nuclear membranes first wrap the exposed region of the segregated chromatin mass that is unoccupied by spindle MTs (called the ‘non-core’ domain) (Liu and Pellman, 2020). The majority of NPCs assemble in the non-core domain to initiate nuclear transport.

After the initial phase of NE formation, BAF accumulates at the ‘core’ domain, which is densely occupied by spindle MTs (Haraguchi et al., 2008). BAF recruits and concentrates LEM-domain proteins, including LEMD2 and emerin, to the core domain, where NE sealing of holes occurs (Haraguchi et al., 2008; von Appen et al., 2020). The LEM-domain protein LEMD2 contributes to NE sealing through its C-terminal winged helix (WH) domain, which directly binds and activates the conserved endosomal sorting complex required for transport (ESCRT)-II/ESCRT-III hybrid protein CHMP7 (Gatta et al., 2021; Gu et al., 2017; von Appen et al., 2020). The WH of LEMD2 copolymerizes with CHMP7 to form 50–100 nm rings *in vitro* and it is thought that the assembly of these rings on the cytosolic surface of NE holes restricts the diffusion of macromolecules (Gatta et al., 2021; von Appen et al., 2020). CHMP7 serves as the NE adaptor for downstream ESCRT-III membrane remodeling machinery, including the spiral filament protein CHMP4B and the MT-severing protein spastin that coordinate spindle disassembly with fusion of small (<100 nm) NE holes (Vietri et al., 2015; Ventimiglia et al., 2018; von Appen et al., 2020). Functions for other LEM-domain proteins at the core domain are less well understood.

The essential role for BAF in NE assembly as well as the functional redundancy of multiple LEM-domain proteins have made it challenging to test whether BAF–LEM binding at the core domain serves a function aside from downstream ESCRT recruitment. In mitotically dividing cells, expression of a dimerization mutant in BAF, deficient in both DNA crosslinking and LEM-domain binding, results in hyper-micronucleation where membranes wrap individual chromosomes because they are not crosslinked (Samwer et al., 2017). Expression of a BAF mutant (BAF-L58R) that selectively prevents binding to LEM-domain proteins did not cause micronucleation, demonstrating that BAF–LEM binding is not essential for the formation of a single nucleus; however, whether sealing during NE formation is impaired in the absence of BAF–LEM binding remains unclear. Expression of BAF-L58R does not support repair of large holes that result from ruptures, indicating that BAF–LEM binding mediates NE sealing in interphase cells (Young et al., 2020). Importantly, CHMP7 accumulates at nuclear rupture sites but is not required for repair of ruptures (Halfmann et al., 2019). Thus, in interphase, BAF–LEM binding has an independent role from CHMP7 in NE sealing, particularly of large NE holes that do not contain MTs (Halfmann et al., 2019; Young et al., 2020). Whether BAF–LEM binding and CHMP7 have separate roles in sealing NE holes that surround spindle MTs is not known. Interestingly, LEMD2 binds MTs directly (von Appen et al., 2020), so there might be a separate function from binding to BAF for LEMD2 with CHMP7 in the presence of MTs.

**Fig. 1.**
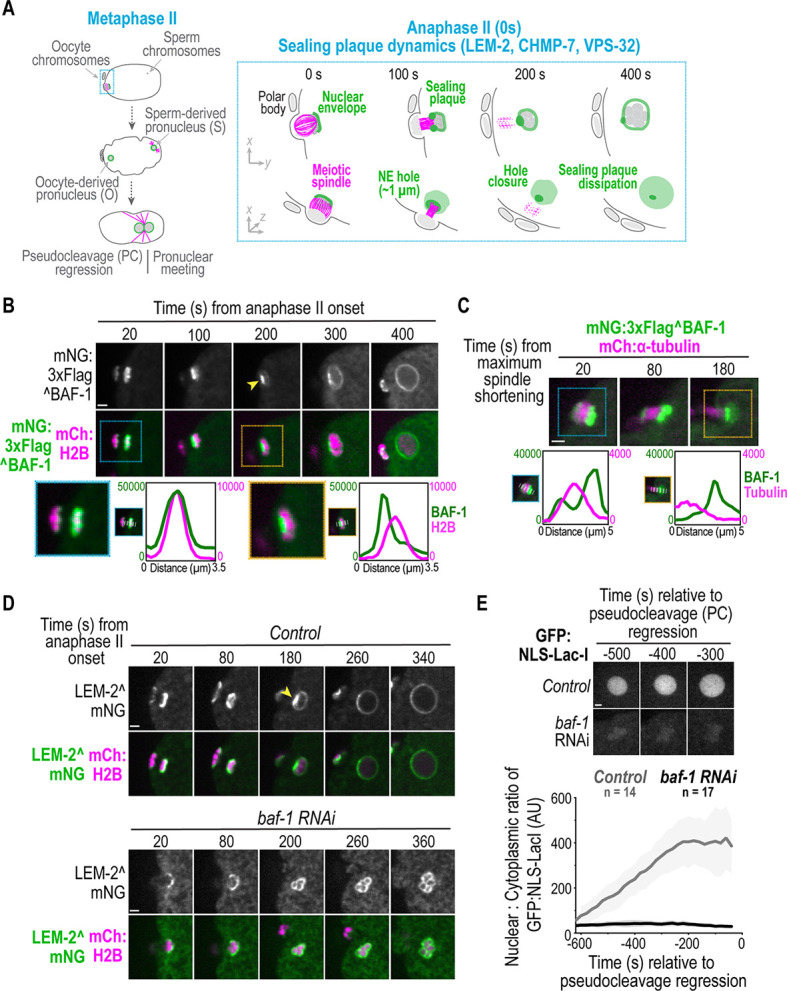
**BAF-1 is required for sealing plaque formation during meiosis II in *C. elegans*.** (A) Left: schematic representation of oocyte- and sperm-derived pronuclear formation and migration after meiosis II. Right (box): schematic of sealing plaque dynamics relative to anaphase II. (B) Spinning-disk confocal images from a time-lapse series of mNG^BAF-1 dynamics relative to anaphase II. The yellow arrowhead marks the sealing plaque. Bottom: zoomed insets of select frames for background-corrected line scans of the markers at the indicated timepoints. Time in seconds relative to anaphase II onset is shown. (C) Spinning-disk confocal images from a time-lapse series of mNG^BAF-1 (green) and meiotic spindle microtubules (magenta). Bottom: zoomed insets of select frames for background-corrected line scans of the markers at the indicated timepoints. Time in seconds relative to maximum spindle shortening is shown. (D) Spinning-disk confocal images from a time lapse series of LEM-2^mNG in the indicated conditions. The yellow arrowhead marks the sealing plaque. Time in seconds relative to anaphase onset is shown. (E) Top: spinning-disk confocal images of GFP:NLS-LacI fluorescence in oocyte-derived pronuclei in the indicated conditions. Bottom: plot (average±s.d.) of normalized nuclear GFP:NLS-LacI fluorescence for the indicated conditions. Time in seconds relative to pseudocleavage (PC) regression is shown. *n* indicates the number of embryos. AU, arbitrary units. Scale bars: 2 μm.

Here, we combined genetic analyses with live imaging of NE formation in *C. elegans* oocytes to determine the shared and unique functions for BAF (BAF-1), LEM-domain proteins and CHMP-7 in NE sealing. Similar to mitotic NE formation, the NE seals around spindle MTs after anaphase of meiosis II in fertilized oocytes. This event establishes the oocyte-derived pronucleus and is the first instance of NE formation for the developing embryo. Using this system, we can also analyze formation of the sperm-derived pronucleus, which occurs in a shared cytoplasm but in the absence of spindle MTs (Penfield et al., 2020; Fig. 1A).

Our previous work defined the assembly dynamics of *C. elegans* LEM-2 (human LEMD2) and presence of CHMP-7 (human CHMP7) at the micrometer-scale sized hole that surrounds the asymmetric meiotic spindle (Penfield et al., 2020). Closure of the post-meiotic NE hole does not require CHMP-7 (Penfield et al., 2020), but whether it requires the accumulation of membrane-bound LEM-domain proteins was not known. The regulation and requirement for BAF-1 in nuclear assembly is conserved in *C. elegans* (Gorjánácz et al., 2007; Margalit et al., 2005). In contrast to human cells, which contain seven LEM-domain proteins, *C. elegans* contains only two integral membrane LEM-domain proteins, EMR-1 (human emerin) and LEM-2, as well as the non-transmembrane LEM-domain protein LEM-3 (human ANKLE1), none of which are essential (Barton et al., 2015; Lee et al., 2000). Combining *lem-2* and *emr-1* mutants resulted in embryonic lethality, which suggested that BAF-1 mediates its essential functions in nuclear assembly through redundant recruitment of these LEM-domain proteins (Liu et al., 2003).

We introduced the conserved BAF-1-L58R separation-of-function mutation at the endogenous locus of *C. elegans baf-1* to disrupt BAF–LEM binding. This mutant background allowed us to analyze the contribution of BAF–LEM binding to NE sealing and assembly. Our work reveals that EMR-1 and LEM-2 function redundantly in binding to BAF-1 to facilitate NE sealing around spindle MTs. We demonstrate that LEM-2 and CHMP-7 are critical to stabilize the NE. LEM-2–CHMP-7 interactions safeguard against failure in NE assembly when BAF–LEM binding is compromised. Our genetic studies further reveal distinct functions for LEM-2 and EMR-1 in the maintenance of the NE permeability barrier and NE assembly. Thus, our work uncovers redundant and distinct roles for multiple key players in NE formation and dissects the relationship between NE sealing and NE stability to support early embryonic development.

## RESULTS

### Endogenous BAF-1 dynamics after chromosome segregation in meiosis II and mitosis

Fertilization by haploid sperm triggers two rounds of meiosis in prophase I-arrested *C. elegans* oocytes to produce the haploid pronucleus as well as two extruded polar bodies (Fig. 1A, left) (Fabritius et al., 2011). The haploid oocyte-derived pronucleus forms as the acentriolar MT spindle elongates between segregated chromosomes after anaphase II (Fig. 1A, right). The NE initially assembles on the side of chromatin that is farthest from the acentriolar meiotic spindle and wraps around chromatin to form a sealing plaque akin to the core domain that surrounds persisting spindle MTs in mitosis (Fig. 1A, 100 s) (Penfield et al., 2020). The plaque condenses as the spindle dissipates (Fig. 1A, 200 s) and then disperses as the pronucleus rapidly expands (Fig. 1A, 400 s). In contrast to the oocyte-derived pronucleus, the haploid sperm-derived pronucleus, at the opposite end of the embryo, does not undergo closure of a large hole around spindle MTs (Fig. 1A, left). Thus, the fertilized *C. elegans* zygote provides the opportunity to directly compare nuclear assembly with and without closure of a large hole around spindle MTs in a shared cytoplasm (Fig. 1A) (Penfield et al., 2020). Furthermore, analyzing the first instance of NE formation allows us to eliminate confounding effects of prior rounds of failed NE assembly. Once formed, the oocyte-derived and sperm-derived pronuclei meet at pseudocleavage (PC) regression and progress to the first mitotic division (Fig. 1A) (Oegema and Hyman, 2006).

We generated a strain with *baf-1* tagged at its endogenous locus with mNeonGreen (mNG) and 3×Flag at the N-terminus (mNG^BAF-1) to monitor BAF-1 dynamics at the sealing plaque (Fig. S1A). Embryo production and viability were unaffected in this strain, suggesting that the fusion protein did not significantly interfere with BAF-1 function (Fig. S1A). mNG^BAF-1 localized on oocyte chromatin at anaphase II onset and transitioned to a bright focus at the sealing plaque as the adjacent meiotic spindle lengthened and dissipated (Fig. 1B,C). mNG^BAF-1 localized uniformly at the nuclear rim ∼400 s following anaphase II onset (Fig. 1B). mNG^BAF-1 associated with sperm chromatin transitioned to a small focus that enriched along the nuclear rim, with similar dynamics as the oocyte-derived pronucleus (Fig. S1B; Movie 1). Thus, universal signaling mechanisms in the shared cytoplasm of the fertilized oocyte triggered by anaphase II control BAF-1 dynamics on chromatin and at the NE.

The LEM-4-like protein (LEM-4L, encoded by *lem-4*; human ANKLE2) is an adaptor for protein phosphatase 2A (PP2A), which dephosphorylates BAF-1. BAF-1 dephosphorylation enhances its chromatin association immediately after mitotic exit (Asencio et al., 2012; Snyers et al., 2018; Marcelot et al., 2021) (Fig. S1C). Reducing *lem-4* by RNAi-mediated depletion to maintain BAF-1 in a phosphorylated state reduced mNG^BAF-1 accumulation on segregated meiotic chromosomes (Fig. S1D) and delayed its accumulation on mitotic chromosomes, as previously reported (Fig. S1E) (Asencio et al., 2012). Thus, LEM-4L-mediated regulation of BAF-1 dynamics in meiosis II is similar to that in mitotically dividing cells.

### Enrichment of LEM-2 and EMR-1 at the sealing plaque in meiosis II requires BAF-1

We tested whether BAF-1 controls the dynamics of LEM-2 and EMR-1 at the reforming NE and sealing plaque. We generated strains with *lem-2* and *emr-1* endogenously tagged with mNG using CRISPR-Cas9 editing. Enrichment of LEM-2^mNG and mNG^EMR-1 at the sealing plaque was similar to that of mNG^BAF-1 and to prior reports for a *lem-2* transgene (Fig. 1D; Fig. S1F) (Penfield et al., 2020). However, they did not accumulate at the sealing plaque in *baf-1* RNAi-depleted embryos and, instead, membranes surrounded individual chromosomes that appeared condensed, resulting in a multi-lobed oocyte pronucleus (Fig. 1D; Fig. S1F; Movie 2). Furthermore, the initial levels of LEM-2-occupied membranes on chromatin during anaphase II onset were significantly reduced in *baf-1* RNAi-depleted embryos (Fig. 1D; Fig. S1G). mNG^EMR-1 had a substantial ER-associated pool, which made comparable measurements unreliable (Fig. S1F). The higher ER/NE ratio of mNG^EMR-1 that we observed (Fig. S1F) was not apparent in prior work using transgenic strains (Askjaer et al., 2002; Morales-Martínez et al., 2015; Pushpa et al., 2013). This difference might reflect the normal localization of EMR-1 driven from the endogenous locus or differences in the position or type of fluorescent protein fusions. Embryos depleted of *baf-1* did not accumulate GFP:nuclear localization signal (NLS)-LacI (hereafter referred to as GFP:NLS reporter) in the nucleus, indicating a failure in NE sealing (Fig. 1E).

Thus, similar to penetrant RNAi-mediated depletion of BAF in mammalian cells exiting mitosis (Samwer et al., 2017), BAF-1 is necessary to form a single nucleus after meiosis II in *C. elegans.* Additionally, BAF-1 directs formation of the sealing plaque containing LEM-2 and EMR-1, which prompted us to use genetic analysis to understand how the recruitment of LEM-domain proteins by BAF-1 contributes to sealing of the large post-meiotic NE hole.

### BAF-1 binding to LEM-domain proteins is required for its NE localization but is not required for viability

**Fig. 2.**
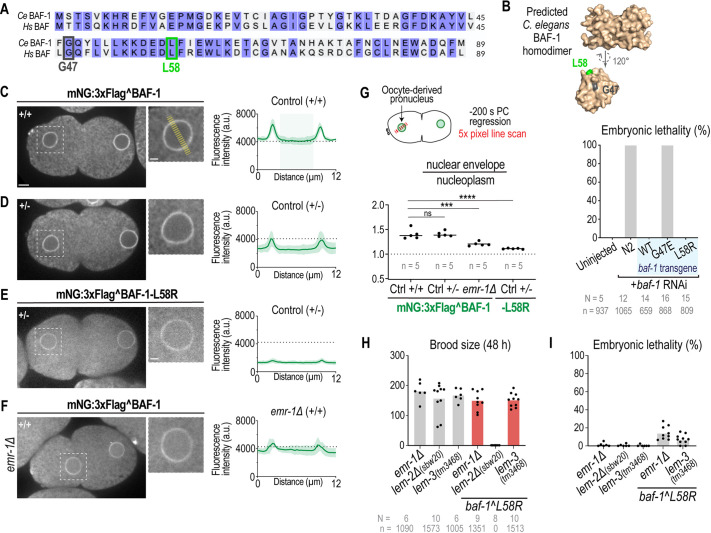
**Selective inhibition of BAF-1 binding to LEM-domain proteins is not essential in *C. elegans.*** (A) Amino acid sequence alignment of human BAF and *C. elegans* BAF-1. Identical amino acid residues are shaded in purple and amino acid residues with separation-of-function mutations are boxed. (B) Top: three-dimensional space-filling model of the predicted *C. elegans* BAF-1 homodimer generated using ColabFold (Mirdita et al., 2022; Goddard et al., 2018; Pettersen et al., 2021) and monomer using AlphaFold Protein Structure Database (Jumper et al., 2021; Varadi et al., 2022). The monomer was rotated 120° on the *x*-axis relative to the dimer, with amino acid residues mutated in this study highlighted. Bottom: plot of percentage of embryonic lethality in the indicated conditions. (C–F) Left: spinning-disk confocal images of mNG^BAF-1 at 200 s prior to PC regression in the indicated conditions. The homozygous (+/+) or heterozygous (+/−) insertion of mNG at the endogenous *baf-1* locus is marked in the images. Right: plots of background-corrected line scans (average±s.d.) of mNG^BAF-1 (*n*=5 embryos). The fluorescence signal from nucleoplasm is shaded in green in control. Scale bars: 5 μm (left panels); 2 μm (zoomed insets). (G) Top: schematic representation of line scan analysis. Bottom: plot representing the ratio of mNG signal at the NE (average of peak signals) and nucleoplasm (average signal between peaks) from line scan analysis in the indicated conditions. Statistical significance was determined by one-way ANOVA and Tukey's post hoc test (ns, not significant; ***P=0.0007; ****P<0.0001). (H,I) Plot (average and replicates) representing brood size (H) and embryonic lethality (I) in the indicated conditions. *N* indicates the number of worms, *n* the number of embryos.

To understand the contribution of BAF–LEM binding to the closure of the large hole that surrounds meiotic spindle MTs, we mutated conserved amino acid residues in *C. elegans* BAF-1 that have been shown to serve as separation-of-function mutations in human BAF (Samwer et al., 2017; Halfmann et al., 2019; Young et al., 2020) (Fig. 2A). BAF-G47E disrupts BAF dimerization and binding to LEM-domain proteins, whereas BAF-L58R selectively inhibits LEM-domain binding (Fig. 2A,B) (Halfmann et al., 2019; Samwer et al., 2017; Young et al., 2020).

We first introduced the mutations into RNAi-resistant *baf-1* transgenes to avoid the embryonic lethality and sterility that could result from significant disruption of *baf-1* function (Fig. S2A). RNAi-mediated depletion of *baf-1* caused 100% embryonic lethality (Fig. 2B), as expected (Gorjánácz et al., 2007; Margalit et al., 2005; Zheng et al., 2000), and this was rescued by the wild-type re-encoded *baf-1* transgene, but not the dimerization-deficient *baf-1(G47E)* mutant transgene (Fig. 2B). Expression of the *baf-1(L58R)* mutant re-encoded transgene in embryos RNAi-depleted of *baf-1* supported viability (Fig. 2B). We therefore introduced the *baf-1(L58R)* mutation in the endogenous *baf-1* locus (Fig. S2B). *baf-1(L58R)* mutant worms expressed normal levels of BAF-1-L58R protein (Fig. S2C), which did not cause embryonic lethality nor sterility, although fertility was partially reduced (Fig. S2D). Thus, BAF–LEM binding is not essential for germline development or embryogenesis.

We next tested whether BAF–LEM binding is required for BAF-1 localization at the INM. BAF localizes to both the nucleoplasm and INM, and LEM-domain proteins bind BAF at the INM (Haraguchi et al., 2007; Liu et al., 2003), so we expected to observe reduced BAF-1-L58R mutant protein at the NE, but not in the nucleoplasm (Halfmann et al., 2019; Samwer et al., 2017; Young et al., 2020). In the one-cell-stage embryo, mNG^BAF-1 was enriched at the NE of the oocyte- and sperm-derived pronuclei, and localized to the nucleoplasm, the ER and diffusely in the cytoplasm (Fig. 2C). Our attempts to create a homozygous *baf-1(L58R)* mutant animal tagged at the endogenous locus with mNG were unsuccessful, suggesting that the mNG tag together with the L58R mutation compromise BAF-1 function at the organismal level. To bypass this issue, we quantified the NE and nucleoplasmic fluorescence signal of heterozygous embryos carrying one copy of mNG-tagged *baf-1* and one untagged copy of wild-type *baf-1* (Fig. 2D). In heterozygous wild-type *mNG^baf-1*/*baf-1* embryos, the NE and nucleoplasmic mNG fluorescence signal was approximately half that of the homozygous *mNG^baf-1* strain (Fig. 2C,D). The NE to nucleoplasmic ratio of the mNG fluorescence was the same in strains homozygous and heterozygous for mNG^BAF1, so even with half the amount of protein, the proportion of wild-type BAF-1 at the NE to that at the nucleoplasm remained constant (Fig. 2G).

The mNG fluorescence levels were lower in heterozygote *mNG^baf-1(L58R)/baf-1* than in wild-type *mNG^baf-1/baf-1* embryos (Fig. 2C–E). The lower NE to nucleoplasmic ratio reflected a greater reduction of the mNG^BAF-1-L58R mutant protein at the NE (Fig. 2E,G). The faint mNG^BAF-1-L58R localization that remains at the nuclear rim might be due to dimerization with untagged wild-type BAF-1 or through association with the nuclear lamina or an unidentified NE adaptor (Holaska et al., 2003; Kono et al., 2022; Samson et al., 2018). The ER-localized signal also appeared reduced in *mNG^baf-1(L58R)*/*baf-1* heterozygote embryos compared to that in *mNG^baf-1/baf-1* embryos, suggesting that BAF-1 might associate with the ER through binding to LEM-domain proteins (Fig. 2C,E).

Reduced nuclear rim signal, but unchanged nucleoplasmic signal, of mNG^BAF-1 in *emr-1Δ* embryos (Fig. 2F,G) is consistent with past findings that EMR-1 contributes to BAF-1 binding at the INM (Asencio et al., 2012). We were unable to assess the localization of mNG^BAF-1-L58R upon deletion of the *lem-2* locus because of the synergistic sterility phenotype that results from this genetic background (see below). We detected lower levels of untagged BAF-1-L58R at the sealing plaque by fixataion and immunostaining (Fig. S2E; *n*=5). However, immunostaining was not reliable in detecting BAF-1 fluorescence across fixed embryos. Importantly, protein levels of untagged BAF-1-L58R in the *baf-1(L58R)* mutant strain that we used throughout this study were unchanged (Fig. S2C). Overall, we conclude that the *C. elegans* BAF-1-L58R mutant is compromised in its NE association, as previously shown for the mutant human BAF homologue (Halfmann et al., 2019; Samwer et al., 2017; Young et al., 2020), and that multiple LEM-domain proteins recruit BAF-1 to the INM.

### Distinct functions for LEM-domain proteins in embryo and germline development revealed by the *baf-1(L58R)* mutant

We crossed the *baf-1(L58R)* strain to mutant alleles in each of the three *C. elegans* LEM-domain genes (*emr-1*, *lem-2* and *lem-3*). We predicted that if LEM-domain proteins have redundant or related functions to BAF–LEM binding, then a double mutant [e.g. *emr-1Δ;baf-1(L58R)*] would result in synergistic phenotypes (e.g. increased lethality). Alternatively, if LEM-domain proteins function only through binding to BAF-1, then a double mutant with *baf-1(L58R)* would not cause a significant increase in embryo viability or production. The *baf-1(L58R)* mutant strain crossed to the *emr-1* deletion or the *lem-3(tm3468)* mutant allele that is reduced in function resulted in only a slight decrease in brood size and embryonic viability (Fig. 2H,I), suggesting that these LEM-domain proteins do not have redundant functions with BAF–LEM binding in embryo and germline development. In contrast, deletion of the *lem-2* gene locus did not cause lethality or sterility on its own (Fig. 2H,I), but the *lem-2Δ(sbw20);baf-1(L58R)* double mutant resulted in 100% sterility (Fig. 2H; Fig. S2G). Note that we found an annotation error in the *lem-2(tm1582)* mutant allele (Fig. S2F), which has been widely used and interpreted as a *lem-2* deletion strain (Barkan et al., 2012; Bone et al., 2014; Dittrich et al., 2012; González-Aguilera et al., 2014; Harr et al., 2020; Morales-Martínez et al., 2015; Penfield et al., 2020; Shankar et al., 2022), in which the 3′ region that encodes for the WH domain is intact (Fig. S2F) (Davis et al., 2022). We therefore generated the *lem-2Δ(sbw20)* allele using CRISPR-Cas9 gene editing to delete the entire *lem-2* gene locus for this study (Fig. S2H,I). Taken together, these data indicate that LEM-2, but not EMR-1 or LEM-3, has a redundant function to BAF–LEM binding in germline development, and further verify that the BAF-1-L58R mutant protein is functionally compromised.

### LEM-2 and EMR-1 dynamics at the reforming NE and sealing plaque require BAF–LEM binding

**Fig. 3.**
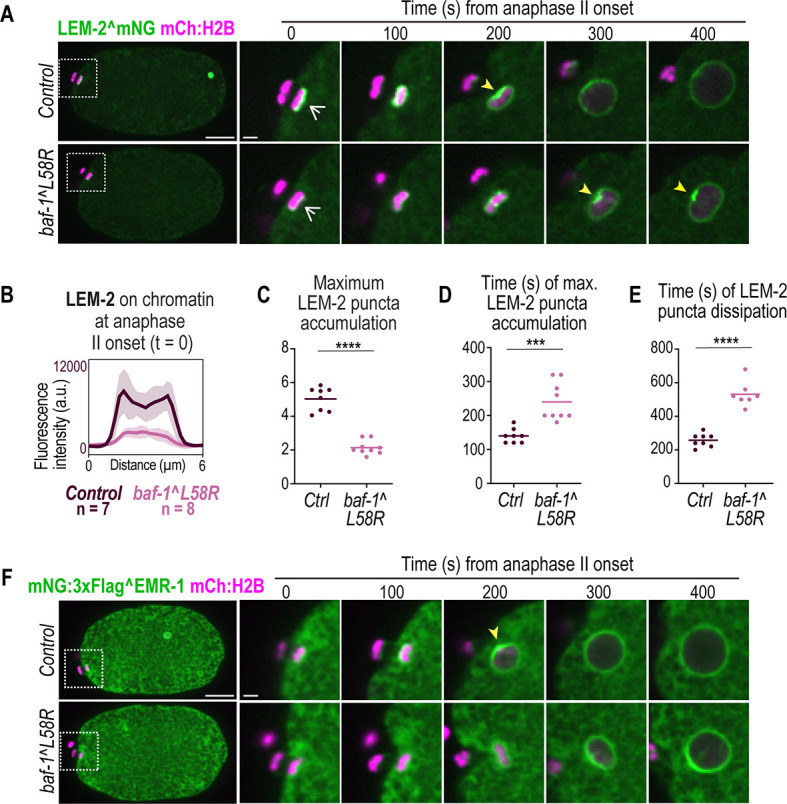
**BAF–LEM interactions orchestrate organized and timely recruitment of sealing proteins during NE formation.** (A) Spinning-disk confocal images from time-lapse series of LEM-2^mNG during oocyte pronuclear formation in the indicated conditions. Yellow arrowheads point to enrichment sites; white arrows highlight the *t*=0 s point. (B) Plot of background-corrected line scan analysis (average±s.d.) of LEM-2^mNG at the nuclear rim at anaphase II onset (white arrows at t=0 s in A) in the indicated conditions. *n* indicates the number of embryos. (C) Plot (average and replicates) representing maximum LEM-2 fluorescence signal at the nuclear rim. (D) Time in seconds relative to anaphase II onset of maximum LEM-2^mNG fluorescence signal in C. (E) Time in seconds relative to anaphase II onset of LEM-2^mNG puncta dissipation. Statistical significance in C–E was determined by unpaired two-tailed Student's *t*-test (**P=0.002; ***P<0.0001; ****P<0.0001). (F) Spinning-disk confocal images from time-lapse series of mNG^EMR-1 in the indicated conditions. Yellow arrowheads point to enrichment sites. Scale bars: 10 μm (whole embryo images); 2 μm (zoomed insets).

We tested whether compromising BAF–LEM protein interactions impacted the recruitment of endogenous LEM-2^mNG and mNG^EMR-1 to the reforming post-meiotic NE (Fig. 3; Fig. S3A). LEM-2^mNG appeared on the nascent NE at anaphase II onset at lower levels in *baf-1(L58R)* mutant embryos compared to those in control embryos (Fig. 3A,B; Movie 3), similar to *baf-1* depletion (Fig. S1G). Instead of the organized sealing plaque that forms directly adjacent to the meiotic II spindle under control conditions (100 s, Fig. 3A), LEM-2^mNG formed smaller foci around condensed chromatin that coalesced into a single punctum with lower fluorescence intensity levels and delayed appearance in *baf-1(L58R)* mutants (Fig. 3C,D). Furthermore, the punctum of LEM-2^mNG in *baf-1(L58R)* mutant oocytes persisted ∼300 s longer than the sealing plaque in control embryos (Fig. 3E). Fully formed oocyte-derived pronuclei in the *baf-1(L58R)* mutant contained lower levels of LEM-2^mNG at the NE (Fig. S3B,C), despite equal levels of global LEM-2 protein (Fig. S3D). Similar observations were made for mNG^EMR-1 dynamics in *baf-1(L58R)* mutants (Fig. 3F; Fig. S3B,C). Additionally, *baf-1(L58R)* mitotic embryos showed delayed LEM-2^mNG on nascent nuclear membranes that did not fully enrich at the core domain (Fig. S3E–G). These data show that BAF-1 recruits and organizes LEM-domain proteins to form the sealing plaque through its BAF–LEM domain binding function. LEM-2 and EMR-1 can localize to the NE independent of BAF-1 binding, albeit at significantly lower levels (Fig. S3B,C), consistent with reports that these proteins have binding partners at the NE other than BAF-1, including lamins and chromatin-regulatory proteins (Caravia et al., 2022; Holaska and Wilson, 2007; Moser et al., 2020).

### LEM-2 and EMR-1 compensate for each other in binding to BAF-1 to seal the NE around spindle MTs

**Fig. 4.**
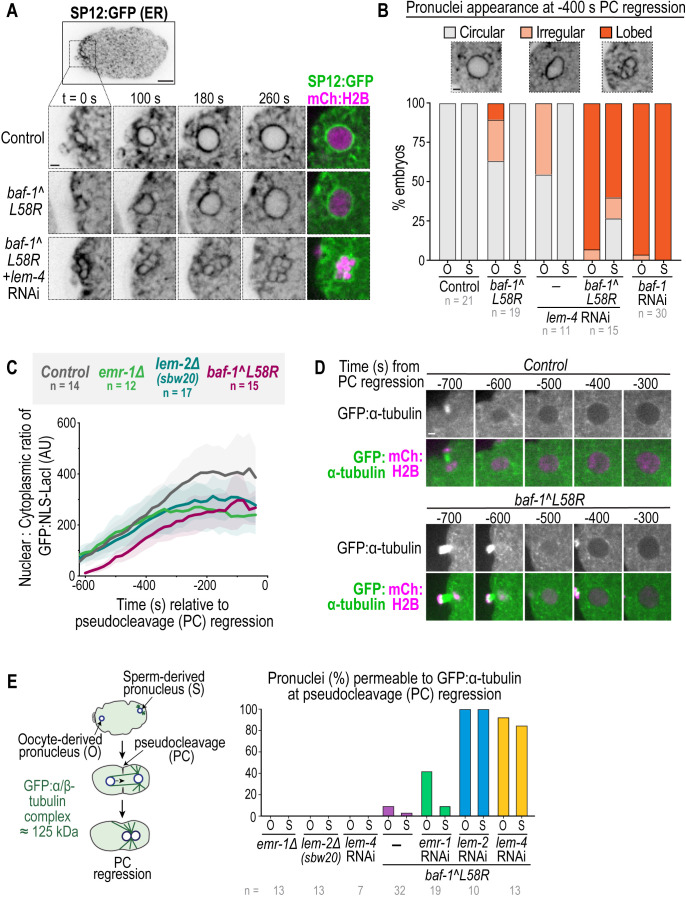
**Reliance on BAF–LEM binding to seal the large NE hole surrounding meiotic spindle microtubules.** (A) Spinning-disk confocal images from time-lapse series of oocyte pronuclear formation marked by SP12:GFP (ER marker) and mCh:histone(H)2B in the indicated conditions. (B) Plot representing the percentage of oocyte-derived (‘O’) and sperm-derived (‘S’) pronuclei categorized as circular, irregular or lobed at −400 s relative to PC regression in the indicated conditions. (C) Plot (average±s.d.) of the ratio of normalized GFP:NLS-LacI fluorescence in the oocyte pronucleus for the indicated conditions relative to PC regression. Statistical significance between control and *baf-1(L58R)* embryos was determined by unpaired two-tailed Student's *t*-test at the time points −500 s (**), −400 s (**) and −300 s (****) relative to PC regression directly after sealing (**P<0.01; ****P<0.0001). (D) Spinning-disk confocal images from time-lapse series of nuclear GFP:α-tubulin during oocyte pronuclear formation in the indicated conditions. Time in seconds relative to PC regression is indicated. (E) Left: schematic that represents the nuclear exclusion of GFP:α-tubulin from pronuclei during pronuclear migration and PC formation and regression. Right: plot representing the percentage of oocyte-derived (‘O’) and sperm-derived (‘S’) pronuclei permeable to GFP:α-tubulin at PC regression. *n* indicates the number of embryos. Scale bars: 2 μm.

We compared NE assembly and sealing in oocyte- versus sperm-derived pronuclei to determine whether there is a unique requirement for BAF–LEM binding in closure of the large NE hole surrounding spindle MTs (Fig. 4). Live imaging of a general ER marker (SP12:GFP) and chromosomes [mCh:histone(H)2B] showed that 100% of both oocyte and sperm-derived pronuclei RNAi-depleted of *baf-1* were multi-lobed (Fig. 4B), whereas in *baf-1(L58R)* mutants a proportion of oocyte- but not sperm-derived pronuclei were malformed and had faint intranuclear membranes (Fig. 4A,B). Reducing *lem-4* levels to prevent dephosphorylation of BAF-1 mildly impacted nuclear shape on its own, but resembled a penetrant BAF-1 depletion in *baf-1(L58R)* mutants, specifically in the oocyte-derived pronucleus (Fig. 4A,B; Movie 4). These data indicate that there is a greater reliance on BAF–LEM binding for nuclear formation when the large hole that surrounds spindle MTs must be sealed. Nuclear formation in mitotic *baf-1-L58R* embryos was delayed and resulted in smaller nuclei (Fig. S4A,B), suggesting that BAF–LEM binding contributes to NE assembly in both meiosis and mitosis in *C. elegans*.

We next monitored the time course of nuclear import of the GFP:NLS reporter following anaphase II onset in *baf-1(L58R)* mutant embryos (Fig. 4C; Fig. S4C). The nuclear to cytoplasmic ratio of GFP:NLS directly following nuclear formation of the oocyte-derived, but not the sperm-derived, pronucleus was significantly lower in *baf-1(L58R)* embryos compared to that in wild-type embryos (Fig. 4C; Fig. S4C). Furthermore, nuclear exclusion of GFP:α-tubulin, which is actively exported from pronuclei (Hayashi et al., 2012) and serves as an indicator of the closure of large NE holes (Penfield et al., 2020), was delayed, but not inhibited, in the *baf-1(L58R)* mutant oocyte-derived pronucleus (Fig. 4D,E). These data indicate that the timely closure of the large NE hole surrounding meiotic spindle MTs requires the ability of BAF to bind LEM-domain proteins.

Deletion of *lem-2* did not impact the initial rate of nuclear import (Fig. 4C) nor nuclear assembly (Fig. S4D,E) following anaphase II, although the nuclear GFP:NLS signal did not reach the maximum wild-type levels in these mutant embryos (Fig. 4C). Similarly, deletion of *emr-1* did not impact the initial rate of nuclear import, but resulted in a lower retention of nuclear GFP:NLS at later time points (Fig. 4C). These data suggested that LEM-2 and EMR-1 are not required for the establishment of the NE permeability barrier on their own but are involved in the maintenance of the NE permeability barrier.

To test whether roles for EMR-1 and LEM-2 in the maintenance of the NE permeability barrier depend on binding to BAF-1, we used RNAi-mediated depletion of either *emr-1* or *lem-2* in *baf-1(L58R)* embryos and assessed nuclear exclusion of GFP:α-tubulin. In *baf-1(L58R)* embryos, RNAi-mediated depletion of *emr-1* resulted in ∼42% of oocyte-derived pronuclei containing nuclear GFP:α-tubulin at PC regression (Fig. 4E), suggesting that EMR-1 has an additional function beyond binding to BAF-1 in stabilizing the NE barrier when NE sealing around spindle MTs is required. Depletion of *lem-2* in *baf-1(L58R)* embryos resulted in 100% of both oocyte and sperm pronuclei failing to exclude GFP:α-tubulin (Fig. 4E) and to form a proper NE, as assessed with a membrane marker (Fig. S4D). These data suggest that LEM-2 and BAF–LEM binding have overlapping functions in NE formation. Both oocyte- and sperm-derived pronuclei were permeable to GFP:α-tubulin upon RNAi-mediated depletion of *lem-4* in the *baf-1(L58R)* mutant (Fig. 4E), further supporting the general requirement for both BAF-1 chromatin association and LEM-domain binding to support stable, nuclear assembly.

We conclude that LEM-2 and EMR-1 compensate for each other in binding to BAF-1 to ensure NE sealing around spindle MTs – loss of either gene alone results in milder defects in NE sealing than in the *baf-1(L58R)* mutant. Compromising BAF–LEM binding further revealed distinct functions for LEM-2 and EMR-1. EMR-1 maintains the NE barrier when hole closure around spindle MTs is faulty, whereas LEM-2 functions redundantly with BAF–LEM binding to ensure stable nuclear assembly.

### NE assembly around spindle MTs relies on CHMP-7 when BAF–LEM binding is compromised

**Fig. 5.**
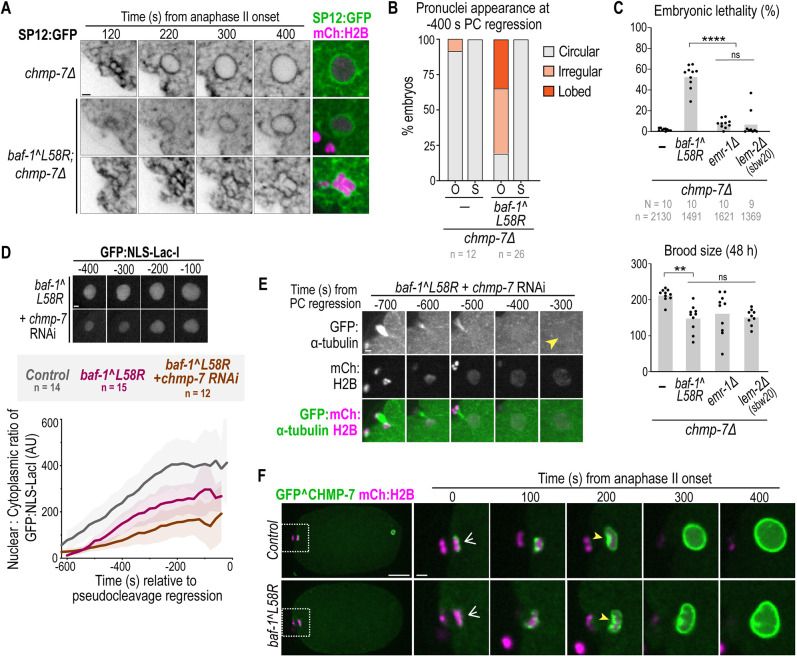
**CHMP-7 maintains nuclear integrity when BAF–LEM-mediated hole closure is compromised.** (A) Spinning-disk confocal images from time-lapse series of SP12:GFP (ER) and mCh:histone(H)2B in the indicated conditions during oocyte-derived pronuclear formation. (B) Plot representing the percentage of oocyte-derived (‘O’) and sperm-derived (‘S’) pronuclei categorized as circular, irregular or lobed at −400 s relative to PC regression in the indicated conditions. (C) Plots (average and replicates) of the percentage of embryonic lethality and brood size in the indicated conditions. Statistical significance was determined by one-way ANOVA and Tukey's post hoc test (ns, not significant; **P=0.0027; ****P<0.0001). (D) Plot (average±s.d.) of normalized nuclear GFP:NLS-LacI fluorescence in the indicated conditions. Time in seconds relative to PC regression is indicated. Control and *baf-1-L58R* data are replicated from Fig. 4C. (E) Spinning-disk confocal images from time-lapse series of GFP:α-tubulin in the oocyte-derive pronucleus in the indicated conditions. Time in seconds relative to PC regression is indicated. The yellow arrowhead marks nuclear GFP:α-tubulin. (F) Spinning-disk confocal images from time-lapse series of mNG^CHMP-7 during oocyte pronuclear formation in the indicated conditions. White arrows denote initial recruitment of GFP^CHMP-7 to chromatin and yellow arrowheads point to enrichment. Time in seconds relative to anaphase II onset is indicated. *N* indicates the number of worms, *n* the number of embryos. Scale bars: 10 μm (whole embryo images); 2 μm (all other images).

The mild NE assembly defects and delayed sealing in the absence of BAF–LEM binding prompted us to test for other factors that may function redundantly to prevent catastrophic loss of the NE permeability barrier. Our data showing that LEM-2 and EMR-1 are recruited to the NE in the absence of BAF–LEM binding suggested that downstream ESCRT-mediated NE remodeling might support NE formation under these conditions. Furthermore, evidence in *C. elegans* suggests that CHMP-7 contributes to NE sealing and stability under conditions of increased lipid biogenesis or a weakened nuclear lamina (Penfield et al., 2020; Shankar et al., 2022). We did not detect defects in the formation of the oocyte- and sperm-derived pronuclei in embryos deleted for *chmp-7* (Fig. 5A,B), similar to our past results (Penfield et al., 2020). In ∼35% of *chmp-7Δ;baf-1(L58R)* double mutant embryos, the oocyte- but not sperm-derived pronucleus appeared lobed (Fig. 5A,B; see also Fig. S5A). These NE assembly abnormalities led to ∼47% of *chmp-7Δ;baf-1(L58R)* embryos containing collapsed oocyte-derived pronuclei at pronuclear meeting, whereas all sperm-derived pronuclei appeared normal (Fig. S5A; Movie 5). Approximately half of *chmp-7Δ;baf-1(L58R)* embryos did not survive to hatching (Fig. 5C), which is reflected in the severe mitotic defects that appeared to compound with subsequent cell divisions (Fig. S5B). The enhanced embryonic lethality with loss of *chmp-7* was specific to the *baf-1(L58R)* mutant background in which both EMR-1 and LEM-2 binding to BAF-1 are compromised because *chmp-7Δ;emr-1Δ* and *chmp-7Δ;lem-2Δ(sbw20)* double-mutant embryos were mostly viable (Fig. 5C). These data further support our findings that binding of BAF-1 to EMR-1 and LEM-2 functions redundantly to promote hole closure and suggest that CHMP-7 has a critical role in NE stability when hole closure is faulty.

Although some *chmp-7Δ;baf-1(L58R)* oocyte-derived pronuclei assembled into a fairly normal shape (Fig. 5A,B, ∼19% ‘circular’), they did not establish or maintain nuclear accumulation of the GFP:NLS reporter (Fig. 5D). Furthermore, the majority (94%) of these pronuclei failed to exclude GFP:α-tubulin (Fig. 5E; Fig. S5C). Thus, CHMP-7 is required for successful establishment of the NE permeability barrier when BAF–LEM-domain binding is impaired. The fact that some oocyte-derived pronuclei assemble a semi-normal morphology under these conditions and ∼47% of embryos survived to hatching (Fig. 4C) suggests that the likelihood of NE assembly failure may depend on the extent of loss of the NE permeability barrier and stability of the NE hole. We conclude that CHMP-7 protects against failure in the NE barrier when NE hole closure around spindle MTs is defective.

### BAF–LEM binding controls the levels and dynamics of CHMP-7 during NE formation and sealing

To understand how CHMP-7 contributes to normal NE sealing and assembly in the absence of BAF–LEM binding, we first monitored the levels, dynamics and organization of endogenously tagged GFP^CHMP-7 at the reforming NE. CHMP-7 is constitutively localized to the nuclear rim and in the nucleoplasm in *C. elegans* embryos (Shankar et al., 2022). In control embryos, nuclear rim-associated CHMP-7 appeared during anaphase II onset, wrapped around chromatin and was enriched at the sealing plaque, similar to LEM-2 (Fig. 5F). GFP^CHMP-7 accumulated in the nucleoplasm ∼200 s after anaphase II onset, concomitant with the establishment of the nuclear permeability barrier (Fig. 5F; Movie 6). In the *baf-1(L58R)* mutant, GFP^CHMP-7 levels were initially lower at the nuclear rim (0 s, Fig. S5D) and discrete CHMP-7 foci formed around condensed chromatin (200 s, Fig. 5F), which coalesced into a disorganized focus rather than a distinct plaque (Fig. 5F). CHMP-7 also localized to the intranuclear membranes observed in assembled oocyte pronuclei of the *baf-1(L58R)* mutant (400 s, Fig. 5F). Lower levels and abnormal dynamics of GFP^CHMP-7 were also observed during NE formation in mitotic embryos of the *baf-1(L58R)* mutant (Fig. S5E,F), similar to LEM-2^mNG.

Thus, the localization and dynamics of endogenous CHMP-7 at the reforming NE after meiosis II and mitosis resemble those of LEM-2 and EMR-1 and are consistent with the role of BAF-1 in recruiting and organizing LEM-domain proteins that then regulate CHMP-7 localization and dynamics. However, the enhanced severity of phenotypes in the *chmp-7Δ;baf-1(L58R)* double mutant suggested that CHMP-7 has functions independent of BAF–LEM binding that ensure NE sealing and assembly.

### LEM-2-dependent and -independent pools of CHMP-7 contribute to post-meiotic NE assembly

**Fig. 6.**
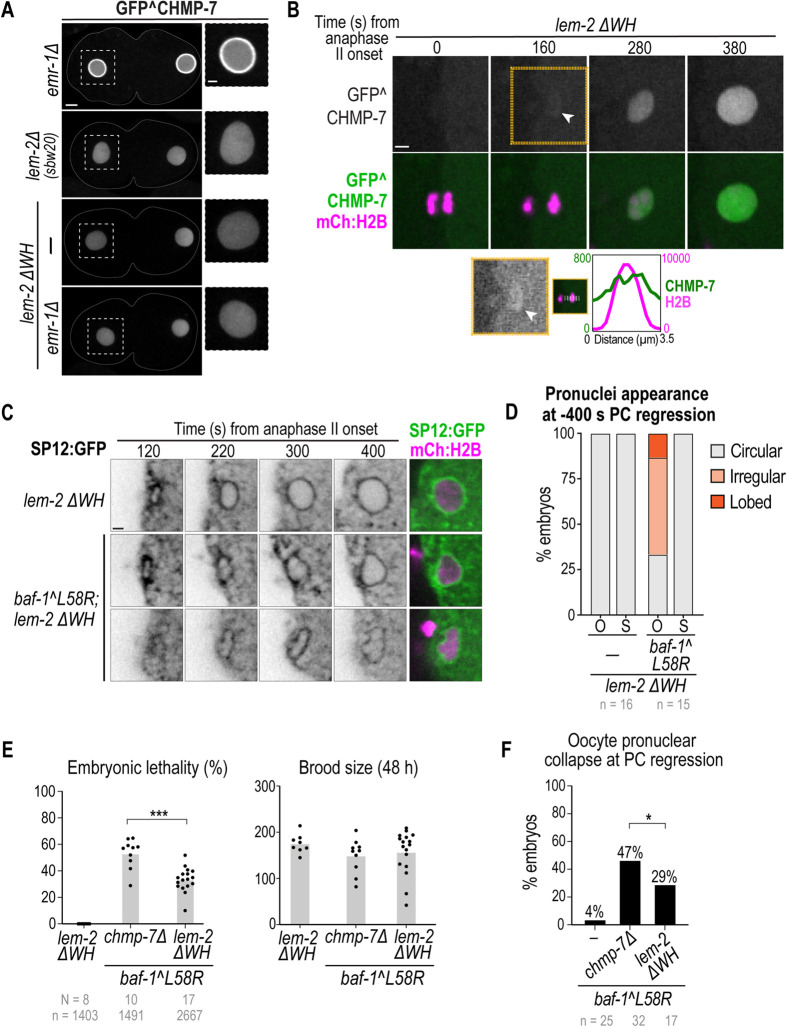
**Compromising BAF–LEM binding reveals a role for CHMP-7 in nuclear stability independent of LEM-2 binding.** (A) Spinning-disk confocal images of GFP^CHMP-7 in embryos at −200 s relative to PC regression in the indicated conditions. Scale bars: 5 μm (left panels); 2 μm (zoomed insets). (B) Spinning-disk confocal images from time-lapse series of GFP^CHMP-7 and mCh:histone(H)2B during oocyte pronuclear formation in *lem-2 ΔWH* embryos. Time in seconds relative to anaphase II onset is indicated. The white arrowhead denotes faint membrane association of GFP^CHMP7 prior to nuclear enrichment. The yellow outlined panel is reproduced below as a montage with brightness and contrast adjusted. The plot represents the background-corrected line scan of the fluorescence of GFP^CHMP-7 and mCh:histone(H)2B across the reforming nucleus at 160 s post anaphase II onset. Scale bar: 2 μm. (C) Spinning-disk confocal images from time-lapse series of the indicated markers in the indicated conditions. Scale bar: 2 μm. (D) Plot representing the percentage of oocyte-derived (‘O’) and sperm-derived (‘S’) pronuclei categorized as circular, irregular or lobed at −400 s relative to PC regression in the indicated conditions. (E) Plots (average and replicates) of the percentage of embryonic lethality and brood size in the indicated conditions. Statistical significance of the indicated conditions was determined by Welch's unpaired two-tailed *t*-test (***P=0.0002). (F) Plot representing the percentage of embryos that underwent oocyte pronuclear collapse in the indicated conditions. Statistical significance of the indicated conditions was determined by Fisher's exact test (*P=0.0130). *N* indicates the number of worms, *n* the number of embryos.

CHMP-7 localizes to the NE in *baf-1(L58R)* mutants during reformation, albeit in a reduced and disorganized manner at initial timepoints. Prior work had suggested that both LEM-2 and EMR-1 are redundantly required for CHMP-7 localization to the INM in *C. elegans* (Shankar et al., 2022); however, the misannotated *lem-2(tm1582)* mutant allele strain was used to assess CHMP-7 localization (see Fig. S2F). We found that, unlike the *lem-2(tm1582)* mutant allele strain (Fig. S6A), nuclear rim localization of GFP^CHMP-7 was not detectable in the CRISPR-Cas9-gene-edited *lem-2Δ(sbw20)* strain (Fig. 6A). GFP^CHMP-7 localization to the ER in mitotically dividing cells was also abolished in *lem-2Δ(sbw20)* embryos (Fig. S6B). Deletion of the WH domain of *lem-2* using CRISPR-Cas9 gene editing further revealed that the WH of LEM-2 is responsible for recruiting CHMP-7 to the nuclear rim, similar to other systems (Fig. 6A; Fig. S6C) (Gu et al., 2017; von Appen et al., 2020). Deletion of *emr-1* in this genetic background did not result in a change in GFP^CHMP-7 localization (Fig. 6A). Thus, LEM-2 is required to retain the majority of CHMP-7 at the INM in *C. elegans* embryos. We did observe a very slight nuclear rim fluorescence signal of GFP^CHMP-7 in *lem-2Δ(sbw20)* and *lem-2ΔWH* mutants ∼160–180 s after anaphase II onset in meiosis and anaphase onset in mitosis (Fig. 6B; Fig. S6D), suggesting that some CHMP-7 can associate with the nuclear rim without LEM-2. Neither LEM-2 nor EMR-1 are necessary for nuclear accumulation of CHMP-7 (Fig. 6A), and we observed increased nuclear GFP^CHMP-7 levels in *lem-2Δ(sbw20)* and *lem-2ΔWH* oocyte-derived pronuclei (Fig. S6E). This suggested that the CHMP-7 pool that does not localize to the nuclear rim in the absence of LEM-2 binding accumulates in the nucleoplasm. Taken together, these data suggest that CHMP-7 localized to the NE in the absence of BAF–LEM binding is recruited by the LEM-2 WH domain, which might explain why CHMP-7 can compensate for defective NE sealing in the *baf-1(L58R)* background.

We tested whether the LEM-2 independent pool of CHMP-7 that is mostly nuclear enriched supports nuclear assembly. Nuclear GFP^CHMP-7 accumulated during post-meiotic and mitotic NE formation but failed to enrich at the sealing plaque or core domain without LEM-2 (Fig. 6B; Fig. S6D; Movie 7). We monitored NE formation in *lem-2ΔWH;baf-1(L58R)* double-mutant embryos and observed that a lower percentage displayed lobed oocyte-derived pronuclei (∼13%; Fig. 6C,D) compared to the *chmp-7Δ;baf-1(L58R)* double mutants (∼35%; see Fig. 5B). These data suggested that nuclear CHMP-7 partially supports NE formation when not bound to LEM-2. Furthermore, the incidence of oocyte pronuclear collapse and embryonic lethality was significantly reduced in the *lem-2ΔWH;baf-1(L58R)* mutants compared to that in *chmp-7Δ;baf-1(L58R)* mutants (Fig. 6E,F). Taken together, these data reveal that LEM-2-independent pools of CHMP-7 contribute to NE formation and provide evidence for a functional nucleoplasmic pool of CHMP-7 in *C. elegans*.

## DISCUSSION

We demonstrate that BAF-1 binding to LEM-domain proteins determines the position and timing of LEM-2, EMR-1 and CHMP-7 assembly at the sealing plaque, which forms the core domain of the NE adjacent to spindle MTs in fertilized *C. elegans* oocytes. We show that NE hole closure around spindle MTs does not depend on LEM-2, EMR-1 or CHMP-7 individually, but BAF binding to LEM-2 and EMR-1 functions redundantly to enable hole closure. Our genetic analysis also revealed unique functions for LEM-2 and EMR-1 aside from binding to BAF-1 in NE stability and germline development. Furthermore, we demonstrate that CHMP-7 becomes essential to the formation of the NE permeability barrier and embryonic viability in the absence of BAF–LEM domain binding. Both LEM-2-dependent and -independent pools of CHMP-7 contribute to this essential function. Thus, multiple redundant mechanisms exist to prevent failure in post-meiotic NE assembly, which is critical for early embryo development.

We propose that BAF-mediated recruitment of LEM-domain proteins and associated membranes resolves large gaps in the core domain of the NE that is obstructed by spindle MTs. CHMP-7 functions with LEM-2 to stabilize this region of the NE against failure in hole closure (Fig. S6F). Our prior work showed that limiting membrane biogenesis ensures successful post-meiotic hole closure (Penfield et al., 2020; Barger et al., 2022), providing further evidence that membrane feeding to narrow and close holes establishes the NE permeability barrier. The function for BAF–LEM binding in post-meiotic hole closure is similar to its role in repair of interphase ruptures that do not contain MTs but are also micrometer-sized (Halfmann et al., 2019; Young et al., 2020). *In vitro* cross-linked BAF can exclude large macromolecules (Samwer et al., 2017), and it is possible that BAF–LEM interactions further serve to plug large NE holes to promote membrane feeding. Our genetic analysis also shows that different LEM-domain proteins serve distinct functions in NE assembly that are independent but partially redundant with BAF–LEM binding.

The LEM-2 WH domain activates CHMP-7 to promote polymerization *in vitro* (Gatta et al., 2021; von Appen et al., 2020). However, neither LEM-2 nor CHMP-7 are required for closure of the large post-meiotic NE hole on their own (Penfield et al., 2020). LEM-2 and CHMP-7 assembly might instead provide a fallback mechanism that stabilizes the NE hole should NE sealing fail (Fig. S6F). A role for CHMP-7 in restricting NE hole size has recently been suggested in *Schizosaccharomyces pombe* (Ader et al., 2023). It is also possible that LEM-2 and CHMP-7 restrict uncoordinated membrane feeding or remodel abnormal membranes at the core domain that otherwise make NE assembly vulnerable to failure, especially with the presence of a large hole and spindle MTs. Evidence in mammalian cells and budding yeast suggests that improper activation and mislocalization of CHMP7 can lead to harmful nuclear membrane deformations (Thaller et al., 2019; Vietri et al., 2020). Thus, the disorganized assemblies of core proteins that we observed in the absence of BAF–LEM domain binding may not only be perturbed in their function for sealing, but also deleterious to NE formation. We also cannot eliminate the possibility that the BAF-1-L58R mutant might retain some binding to LEM-domain proteins, although the accumulation of LEM-domain proteins at the NE is significantly reduced. Nevertheless, our genetic evidence showing that loss of CHMP-7 exacerbates phenotypes in the BAF-1-L58R mutant suggests that these assemblies or other pools of CHMP-7 (see below) protect against assembly failure.

In *C. elegans*, CHMP-7 is constitutively localized in the nucleoplasm and at the INM in fully formed nuclei (Shankar et al., 2022). This localization is unlike that of CHMP7 in budding yeast and mammalian cells, in which it is associated with the cytoplasm or ER, respectively, and thus physically segregated from LEM-2, presumably in an inactive form (Bauer et al., 2015; Chu et al., 2023; Gatta et al., 2021; Olmos et al., 2016; Thaller et al., 2019). CHMP7 is excluded from the nucleus in these systems through a conserved nuclear export signal and activated when it gains access to the LEM-2 WH during mitosis and upon loss of NE integrity in interphase (Gatta et al., 2021; Thaller et al., 2019; Vietri et al., 2020). We discovered that LEM-2 is required for the majority of CHMP-7 to associate with the INM in *C. elegans*. The fact that CHMP-7 is nuclear enriched and therefore always accessible to LEM-2 in *C. elegans* highlights that there might be alternative mechanisms that activate the LEM-2–CHMP-7 complex upon loss of NE integrity. It is also possible that LEM-2 and CHMP-7 are constitutively co-polymerized at the INM but are limited in their ability to recruit downstream ESCRTs until there is a breach in the NE.

We found that a LEM-2-independent pool of CHMP-7 partially supports NE assembly (Fig. S6F, bottom panel). Whether the nuclear or minor nuclear rim pool of CHMP-7 is the functional pool that partially supports NE assembly is unclear. Prior work showed that CHMP7 binds peripherally to membranes through a hydrophobic patch that, in budding yeast, resembles an amphipathic helix and this association is necessary for its function (Olmos et al., 2016; Thaller et al., 2021). Prediction algorithms were unable to identify a reliable amphipathic helix in a similar region for *C. elegans* CHMP-7 where a stretch of hydrophobic residues exist. It remains possible that EMR-1 has a role in recruiting a very minor pool of CHMP-7 to the NE. Interestingly, aberrant nuclear accumulation of CHMP7 is associated with diseased neurons from patients with amyotrophic lateral sclerosis and reduces NPC levels (Baskerville et al., 2023; Coyne et al., 2021). There might be aspects of *C. elegans* CHMP-7 that allow it to perform a unique function in the nucleus when unbound to membranes.

It is surprising that some oocyte-derived pronuclei assemble in the absence of both BAF–LEM domain binding and CHMP-7 even with a defective nuclear permeability barrier. This suggests that nuclear assembly is prone to failure at a stochastic rate that might depend on whether the timing of dissolution and detachment of spindle MTs is synchronized with the local stability of the NE hole. NE irregularities may sometimes prevent nuclear assembly or allow the assembly of nuclei that later collapse. Our genetic experimental system of *C. elegans* allowed us to quantitively analyze NE sealing and monitor its impact on nuclear assembly and embryonic survival. Together, the redundant mechanisms that support NE assembly, made evident in this study, emphasize the robust nature of NE formation that is required for early development.

## MATERIALS AND METHODS

### Strain maintenance and generation

The *C. elegans* strains used in this study are listed in Table S1. Strains were maintained at 20°C on nematode growth media (NGM) plates seeded with OP-50 *Escherichia coli.* The original MosSCI strain was maintained at 15°C.

#### MosSCI strains

The re-encoded *baf-1* transgene was cloned into pCFJ151 (Frøkjaer-Jensen et al., 2008) for integration into the Mos1 site on chromosome I (MosSCI) (Frøkjaer-Jensen et al., 2008). Mutagenesis was performed using the primers listed in Table S2. Injection mixes contained the following: *baf-1* transgene-containing plasmid (10 ng/μl; this study), a plasmid encoding the Mos1 transposase (Pglh-2::transposase, pJL43.1, 10 ng/μl; Frøkjaer-Jensen et al., 2008) and three plasmids encoding fluorescent co-injection markers: Pmyo-2::mCherry (pCFJ90, 1.4 ng/μl; Frøkjaer-Jensen et al., 2008), Pmyo- 3::mCherry (pCFJ104, 2.9 ng/μl; Frøkjaer-Jensen et al., 2008) and Prab-3::mCherry (pGH8, 5.7 ng/μl; Frøkjaer-Jensen et al., 2008). The injection mix was spun down (20,000 ***g***, 15 min, 4°C) to remove particulates. Young adult hermaphrodites (EG8078) were injected and singled onto OP-50-seeded plates for recovery. After ∼7–10 days, non-*unc* progeny lacking fluorescent markers were isolated and screened for transgene integration by PCR.

#### CRISPR-Cas9 deletion strain

The deletion for *lem-2* (W01G7.5) was generated using two CRISPR guides or ‘crRNA’, which were chosen using the custom CRISPR guide algorithm by Integrated DNA Technologies (https://www.idtdna.com/site/order/designtool/index/CRISPR_CUSTOM) (see Fig. S2G). Individually, 1 μl of the purified crRNAs was annealed to 1 μl of trans-activating crRNA (tracrRNA) by incubating RNAs at 95°C for 5 min. A dpy-10 crRNA was used for co-CRISPR selection. An injection mix with the following components was set up at room temperature and incubated for 5 minutes: *lem-2* crRNA-1 (11.7 μM), *lem-2* crRNA-2 (11.7 μM), purified Cas9-NLS protein (QB3 Berkeley, 14.7 μM) and *dpy-10* guide (3.7 μM). Finally, a *dpy-10* repair template (29 ng/ml) (Paix et al., 2015) was added and the mix was spun down (20,000 ***g***) for 30 min at 4°C. The RNA–protein mix was injected into the gonads of N2 young adult worms, which were allowed to recover for 3 days. F1 progeny with a roller phenotype were singled out to individual plates. After 3 days, F1 mothers were genotyped by PCR. The deletion strain was sequenced and outcrossed six times to N2 worms before use and characterization.

#### CRISPR-Cas9 fluorescent knock-in strains

Fluorescent endogenous tagging of *baf-1*, *lem-2* and *emr-1* was performed using a self-excising cassette (SEC) repair template (Dickinson and Goldstein, 2016; Hastie et al., 2019) (see Figs S1A and S3A; Table S3). Homology arms (500–800 bp) were cloned into the SEC vectors [pDD268 (Addgene, #132523) or LL-mNG (Hastie et al., 2019)]. Unique sgRNAs were cloned into the Cas9 guide plasmid pDD122 (Addgene, #47550). Following sequencing, injection plasmids were miniprepped using PureLink HiPure Plasmid Miniprep Kit (Thermo Fisher Scientific). Plasmids were combined at 100 ng/μl SEC plasmid and 50 ng/μl Cas9 plasmid and spun down (20,000 ***g***) for 30 min at 4°C. Young adult N2 worms were injected and rescued to individual plates to recover for 3 days at 25°C. Plates were screened for roller progeny and positive plates were treated with approximately 200–300 μl of hygromycin B (20 mg/ml; Thermo Fisher Scientific). Hygromycin B-resistant roller worms were singled to individual plates to assess roller progeny. Plates that had roller progeny were heat shocked at 34°C for 4 h and allowed to recover at 20°C. Non-roller worms were singled to individual plates and progeny were screened for fluorescent protein integration by microscopy. Knock-in worms were sequence confirmed and outcrossed four times to N2 worms. Of note, N-terminal tagging of LEM-2 was found to reduce protein functionality. N-terminal tagging of EMR-1 produced no discernible issues in protein function.

### RNAi

Primers were designed using Primer3T (https://primer3.ut.ee/) to amplify a 200–1000 bp region of the gene of interest (see Table S2) using N2 gDNA as a template. The amplicon was then column purified and reversed transcribed using a T7 enzyme (MEGAscript, Life Technologies). The synthesized RNAs were purified using phenol–chloroform and resuspended in 1× soaking buffer (32.7 mM Na_2_HPO_4_, 16.5 mM KH_2_PO_4_, 6.3 mM NaCl and 14.2 mM NH_4_Cl). RNA reactions were annealed at 68°C for 10 min, followed by 37°C for 30 min. Double-stranded RNAs (dsRNAs) were brought to a final concentration of ∼2000 ng/μl and stored as 2-μl aliquots at −80°C. For each experiment, a fresh aliquot was diluted to ∼1000 ng/μl using 1× soaking buffer and centrifuged at 20,000 ***g*** for 30 min at 4°C. 0.35 μl of the diluted dsRNA was loaded into the back of a pulled capillary needle and injected into the gut of L4 worms. Injected worms were rescued to plates seeded with OP-50 and allowed to recover prior to imaging or lethality analysis. Knockdown time for the following targets was as follows: *baf-1* (48–72 h), *emr-1* (24 h), *lem-2* (24 h), *lem-4* (24 h) and *chmp-7* (28 h).

### Lethality and brood size quantification

L4 worms were singled (uninjected) or injected with the indicated dsRNAs and allowed to recover for 24 h at 20°C. Worms were then singled to individual plates for 24 h (day 1), and transferred to another plate for a final 24 h (day 2). Worms were then killed and plates corresponding to 24–48 h post injection (day 1) were counted for hatched larvae and unhatched embryos. The next day, the plates corresponding to 48–72 h post injection (day 2) were counted. The total number of embryos and larvae were combined for each time window to calculate the brood size and the 48–72 h embryonic lethality is reported.

### Immunoblotting

#### Generation of whole-worm lysate

Prior to lysate generation, L4 worms were selected 24 h prior. For each sample, a microcentrifuge tube was filled with 30 μl of M9 buffer (22 mM KH_2_PO_4_, 19 mM NH_4_Cl, 48 mM Na_2_HPO_4_, 9 mM NaCl) and the volume was marked. 35 adult worms were moved to an indented slide filled with 75 μl of M9 buffer containing 0.1% Triton X-100. Worms were then collected into the marked microcentrifuge tube and washed three times with M9 containing 0.1% Triton X-100 (200 ***g***, 2 min). After the final wash, samples were brought up to a final volume of 30 μl using M9 containing 0.1% Triton. Then, 10 μl of 4× Laemmli sample buffer was added and the tubes were mixed. The samples were then sonicated at 70°C for 15 min, followed by incubation for 5 min at 95°C. Samples were re-sonicated at 70°C for an additional 15 min. Worm lysates were stored at −20°C until they were run on an SDS-PAGE protein gel.

#### Gel electrophoresis

Worm lysates were loaded on 4–20% Mini-Protean TGX Precast Gels (Bio-Rad). Gels were run at 120 V for 15 min to fully collapse samples and then at 180 V. Transfer to PVDF membranes (Thermo Fisher Scientific) was performed at 4°C at 350 mA for 1.25 h. Membranes were blocked in 5% non-fat milk in TBS containing 0.1% Tween-20 (TBST) for 1 h at room temperature and incubated overnight at 4°C with the following primary antibodies diluted in blocking reagent: 1 μg/ml mouse anti-α-tubulin (DM1A, EMD Millipore), 1 μg/ml rabbit anti-LEM-2 (Novus Biologicals, 48540002), 1 μg/ml rabbit anti-CHMP-7 (Shankar et al., 2022) and 1 μg/ml rabbit anti-BAF-1 (Gorjánácz et al., 2007). The following day, membranes were briefly rinsed in TBS, followed by three 5-min washes in TBST. Membranes were then incubated with appropriate secondary antibodies for 1.25 h at room temperature. Horseradish peroxidase (HRP)-conjugated goat-anti-rabbit (G-21234) and HRP-conjugated goat-anti-mouse (G-21040) antibodies (Thermo Fisher Scientific) were diluted to 1:10,000. Membranes were again briefly rinsed in TBS followed by three 5-min washes in TBST. Membranes were incubated with Clarity Max Western ECL Substrate (Bio-Rad) for 5 min before imaging (BioRad ChemiDoc MP Imaging Systems). Antibody specificity was validated by protein knock-out strains or protein depletion experiments.

### Immunofluorescence

#### Slide preparation

Microscope slides (Fisher Scientific Premium Microscope Slides Superfrost) were coated with poly-L-lysine (1 μg/ml) and dried on a heat block. Slides were then baked at 95°C for 30 min and used the same day.

#### Fixation and immunofluorescence

Fifteen to 20 adult worms were picked into a 4 μl drop of ddH_2_O and covered with a standard 18×18 mm coverslip. Embryos were pushed out of the adult worms by pressing down on the corners of the coverslip with a pipet tip. To crack the eggshell and permeabilize the embryos, slides were placed in liquid nitrogen for ∼5 min. Coverslips were quickly removed using a razor blade. Slides were then fixed in pre-chilled 100% methanol at −20°C for 20 min. Following fixation, slides were washed two times in 1× PBS at room temperature for 10 min each using a coplin jar. After the second wash, samples were blocked with 1% BSA in PBS per slide in a humid chamber for 1 h at room temperature. Slides were then incubated overnight at 4°C with primary antibodies diluted in PBS (45 μl per slide; rabbit anti-LMN-1 or rabbit anti-BAF-1, 1 μg/ml) (Penfield et al., 2018; Gorjánácz et al., 2007). Following primary antibody incubation, slides were washed two times in 1× PBS containing 0.2% Tween 20 (PBST) at room temperature for 10 min each using a coplin jar. Following the second wash, slides were incubated at room temperature for 2 h in the dark with secondary antibodies [anti-rabbit Cy3/Rhodamine, 1:200, AB_2338006; anti-mouse FITC, 1:200, AB_2338599 (Jackson Immunoresearch)] diluted in PBS. Slides were again washed two times in PBST at room temperature for 10 min each in the dark. Samples were stained with 1 µg/ml Hoechst (diluted from a 1 mg/ml stock in H_2_O) for 10 min. Slides were washed quickly once with PBS at room temperature prior to mounting. Mounting medium (Molecular Probes ProLong Diamond Antifade Reagent) was added to each sample and coverslips were adhered with clear nail polish. Slides were dried at room temperature overnight and stored at −20°C.

### Microscopy

#### Live-cell imaging

2% agarose imaging pads were made by sandwiching molten agarose (95°C) on a glass slide. Gravid adult hermaphrodites were dissected using G10 beveled needles in 7 μl of Egg Salts (88.5 mM NaCl, 30 mM KCl, 2.55 mM MgCl_2_, 2.55 mM CaCl_2_ and 3.75 mM HEPES pH 7.4) on a glass slide. Select embryos were transferred to the imaging pad using a mouth pipette. Stage-of-interest embryos were delicately positioned using an eyelash tool and a glass coverslip was gently added on top of the imaging pad. Imaging was performed on an inverted Nikon (Melville, NY) Ti microscope with a 60× (1.4 NA) Plan Apo objective lens, a confocal scanner unit (CSU-XI, Yokogawa) with solid state 150 mW, 488 nm and 100 mW, 560 nm lasers, and an ORCA R-3 Digital CCD Camera (Hamamatsu). For most experiments, images were acquired every 20 s at five 2 μm-step *z*-slices. Imaging was performed in a temperature-controlled room at 20°C.

#### Live-cell imaging of meiosis

Early embryo imaging, prior to eggshell formation, has been previously described (Maddox and Maddox, 2012). In brief, a circle of Vaseline is drawn on a 24×50 mm coverslip (Fisherbrand). 3 μl of Egg Salts is added to the center of the circle to dissect embryos from gravid adults. A second long coverslip is placed on top of first coverslip to form a droplet. Embryos are imaged in the suspended droplet with the Vaseline preventing any harmful compression.

#### Fixed-embryo imaging

Immunofluorescence imaging of fixed embryos was conducted on an inverted Nikon Ti2 Eclipse microscope equipped with solid-state 405, 445, 488, 515, 594, 561, 594 and 640 nm lasers, a Yokogawa CSU-W1 confocal scanner unit, a 60× (1.4 NA) Plan Apo objective lens and a Prime BSI sCMOS camera (Photometrics).

### Image analysis

#### Nuclear import analysis

To determine the fluorescence intensity of GFP:NLS-LacI inside the nucleus of one-cell-stage embryos, the chromatin was traced with either the freehand or circle tool in ImageJ. The camera background was determined by drawing a 50×50 pixel box in vacant areas of the time lapse. Average cytoplasmic values were determined by drawing a 20×20 pixel box inside the embryo, away from the growing or moving nucleus. The nuclear to cytoplasmic ratio was determined by subtracting the average camera background from both nuclear and cytoplasmic values and then dividing the nuclear values by the cytoplasmic values. To account for differences in nuclear size, the ratio was then multiplied by the nuclear area. The import of the GFP:NLS-LacI in to the pronuclei was graphed relative to PC regression.

#### Line scan analysis of proteins at the NE and nucleoplasm

A 5-pixel wide line was drawn across the entire nucleus to determine the fluorescence intensity in ImageJ. Line scans (14 μm) along the oocyte-derived pronucleus were drawn at 200 s prior to PC regression. Lines were drawn to avoid internal membranes to gather isolated nucleoplasmic values. The same line scan was used to acquire the average intensity for the camera background and this average was subtracted from all values. These values were then plotted against the relative position along the line. The two maximum peaks of the NE values were averaged for the ‘NE’ value. ∼15–25 values were averaged in the nucleoplasmic area to represent the nucleoplasm value. The averaged nuclear envelope value was divided by the nucleoplasmic value to calculate the NE/nucleoplasmic ratio.

#### Line scan analysis of NE protein dynamics during meiosis and mitosis

A 3-pixel-wide by 5-μm-long line was drawn on the reforming NE in ImageJ. Anaphase II onset was defined as the start of anaphase B with visible separation of chromatin away from the second polar body at the cortex. Line scans of nascent membranes at anaphase II onset were drawn along chromatin mass using the line or freehand tool in ImageJ. The same line scan was used to acquire the average intensity for the camera background and this average was subtracted from all values. These values were then plotted against the relative position along the line. Line scans of the sealing plaque or puncta enrichment were drawn through the enrichment to the opposite nuclear membrane using the line tool. The maximum peak values of the sealing plaque were divided by the values at the opposing nuclear rim to calculate the ‘LEM-2 puncta accumulation’ value. A similar technique was used to track sealing plaque proteins during mitosis. The freehand line tool was used to draw a 3-pixel-wide line over one face of the nuclear membrane alongside chromatin over time. These values were background subtracted using an averaged camera background value. Five values at each end of the line, representing the non-core regions, were averaged. The maximum-intensity values along the line were divided by the non-core average to track core-domain enrichment over time (see Fig. S3E).

### Statistical analysis

All statistical tests were performed using GraphPad Prism 9. Statistical analysis was performed on datasets with multiple samples and from independent biological repeats. Statistical tests used, sample sizes, definitions of replicates (*N*, *n*), and *P* values (*P*<0.05 as the significance cutoff) are reported in figures, figure legends and text.

## Supplementary Material

10.1242/joces.261385_sup1 Supplementary information

## First Person

This article has an associated First Person interview with the first author of the paper.
