# Supplementary material for: Nuclear envelope assembly relies on CHMP-7 in the absence of BAF–LEM-mediated hole closure
Source: J Cell Sci. 2023 Nov 13;136(21):jcs261385. doi: 10.1242/jcs.261385 (PMC10668030; doi:10.1242/jcs.261385)
Supplement: Supplementary information [file joces-136-261385-s1.pdf]

Barger et al. Figure S1

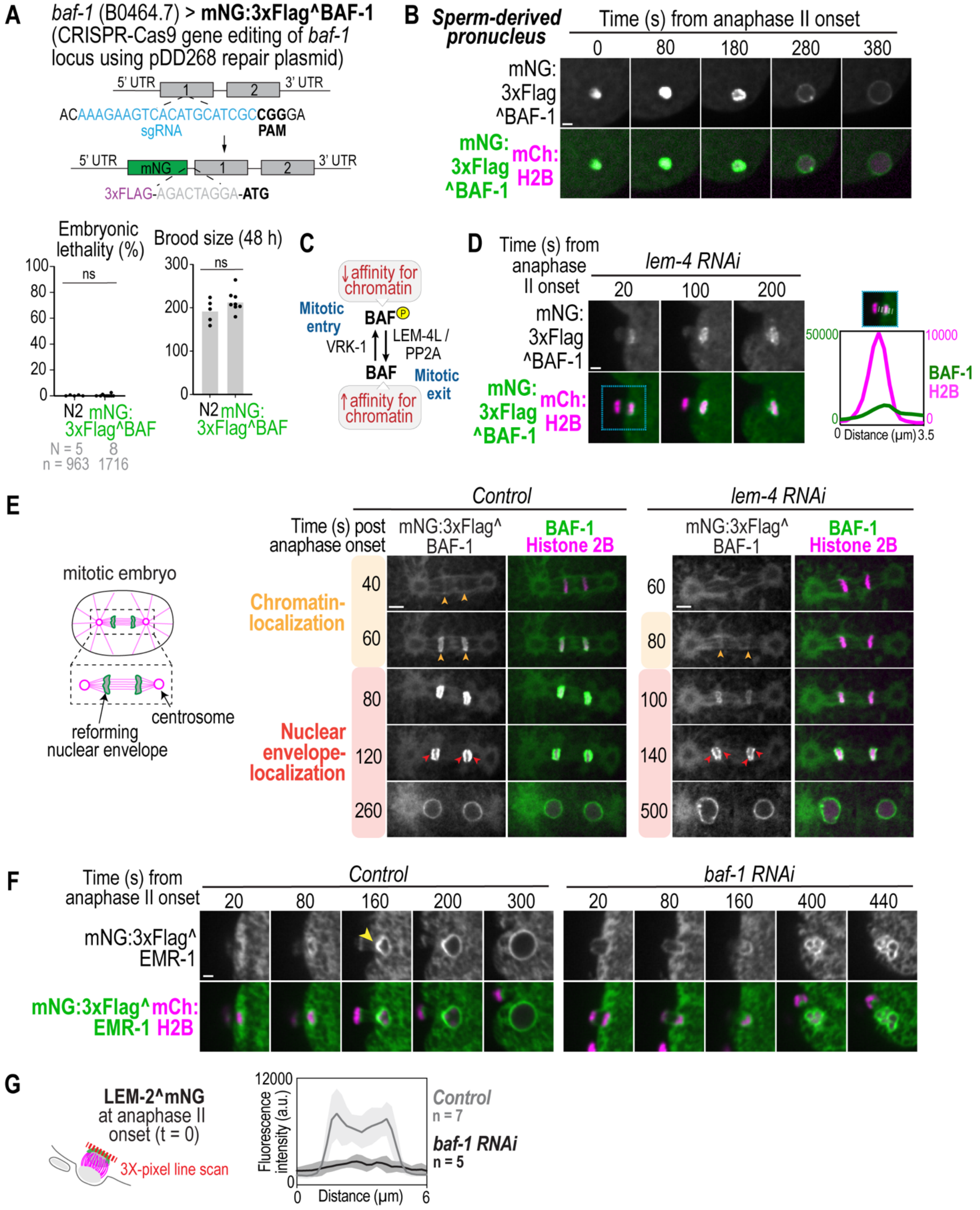

Journal of Cell Science • Supplementary information

Fig. S1. Regulation of BAF-1 dynamics, related to Figure 1. (A) Top, schematic representation of endogenous *baf-1* locus with CRISPR guides used to insert mNG. Below, plot representing percentage of embryonic lethality and brood size in indicated conditions. Statistical significance determined by unpaired Student's t-test (ns, not significant). N = # of worms, n = # of embryos. (B) Spinning disk confocal images from time lapse series of endogenous mNG^BAF-1 localization on sperm-derived pronucleus. Time in seconds relative to anaphase II onset. (C) Schematic of phosphoregulation of BAF-1 and the effect on chromatin interaction. (D) Spinning disk confocal time lapse images of mNG^BAF-1 and mCh:Histone(H)2B with *lem-4* RNAi in oocyte meiosis II. Time is in seconds relative to anaphase II. Right, plot of background-corrected line scan of indicated markers. (E) Left, schematic representation of reforming nuclear envelopes after first mitotic division in *C. elegans*. Right, spinning disk confocal time lapse series of mNG^BAF-1 and mCh:Histone(H)2B in indicated conditions. Color of arrowheads correspond to colored labels. Time in seconds relative to mitotic anaphase onset. Scale bar, 5 µm. (F) Spinning disk confocal images from time lapse series of mNG^EMR-1 dynamics during oocyte-derived pronuclear formation in indicated conditions. Yellow arrow marks sealing plaque. Time in seconds relative to anaphase II onset. Scale bar, 2 µm. (G) Left, schematic of (Right) line scan analysis (average ± SD) of LEM-2^mNG at nuclear rim at anaphase II onset in indicated conditions. n = # of embryos.

Journal of Cell Science • Supplementary information

Barger et al. Figure S2

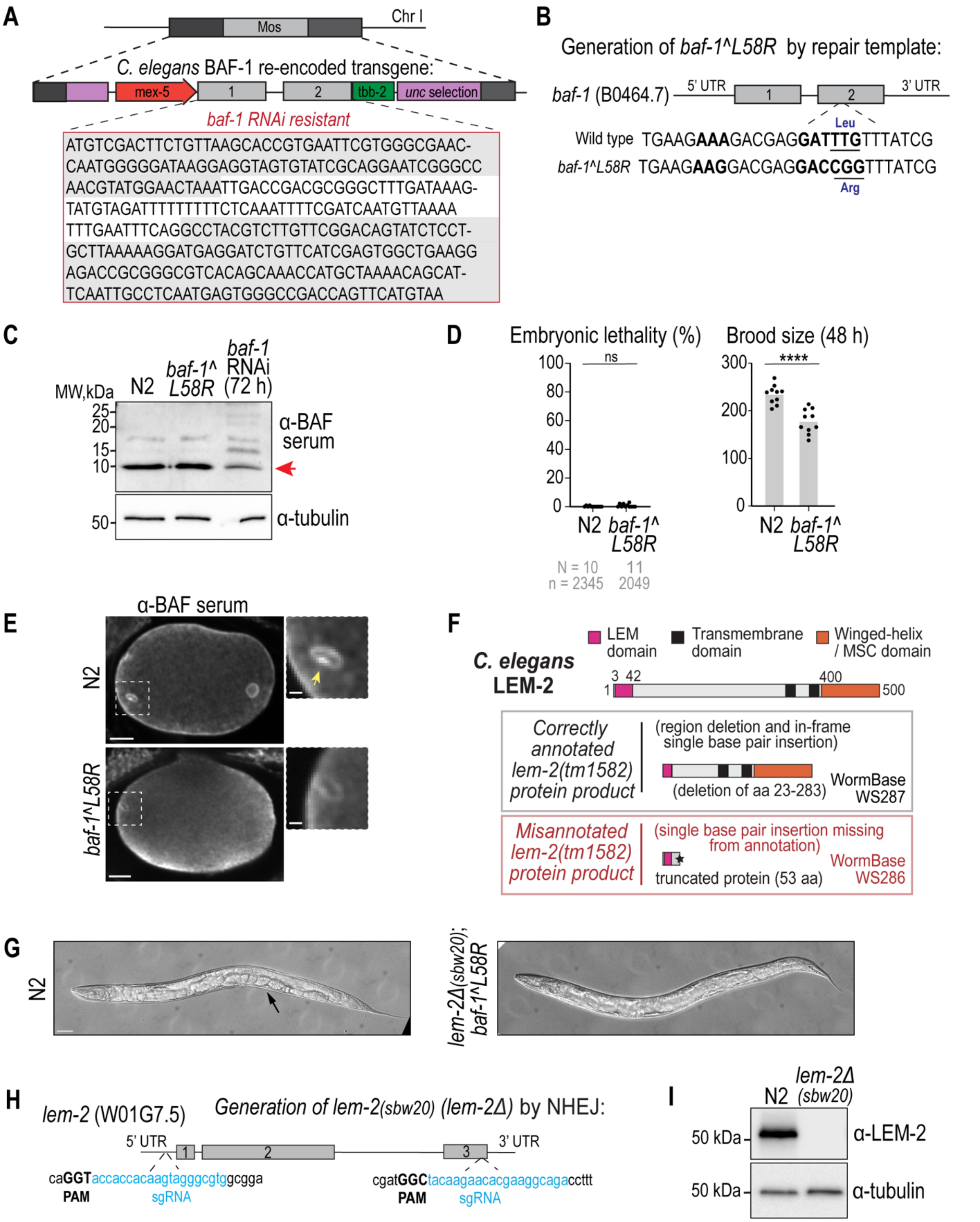

Journal of Cell Science • Supplementary information

Fig. S2. Generation and analysis of *baf-1* and *lem-2* mutant alleles. (A) Schematic showing single copy insertion of the RNAi-resistant *baf-1* transgene integrated in the MosI transposon site of chromosome I. (B) Schematic of CRISPR-Cas9 edits at endogenous *baf-1* locus to generate the *baf-1(L58R)* mutant allele. (C) Representative immunoblot of whole worm lysates incubated with antibodies against BAF in indicated conditions. N = 3 independent experiments. (D) Plots (average + replicates) representing embryonic lethality and brood size in indicated conditions. Statistical significance determined by unpaired Student's t-test (**** = $p < 0.0001$, ns, not significant). N = # of worms, n = # of embryos. (E) Confocal images of *C. elegans* embryos finishing meiosis immunostained with anti-BAF serum in indicated conditions. Yellow arrow points to BAF-1 sealing plaque enrichment. Scale bars, 5 µm; Scale bar in zoom insets, 1 µm. (F) Schematic representation of LEM-2 protein domain structure. Gray box, LEM-2 mutant protein produced from correctly annotated *lem-2(tm1582)* mutant allele. Red box, LEM-2 mutant protein predicted from misannotated *lem-2(tm1582)* mutant allele. (G) Representative brightfield image of whole worms in indicated strain. Black arrow points to germline/embryos. Scale bar, 50 µm. (H) Schematic representation of endogenous *lem-2* locus and CRISPR guides to excise the *lem-2* gene to generate the null allele used in this study. (I) Immunoblot of whole worm lysates incubated with antibodies made against N-terminus (aa 1-100) of LEM-2 protein (Novus Biologicals) in indicated strains.

Barger et al. Figure S3

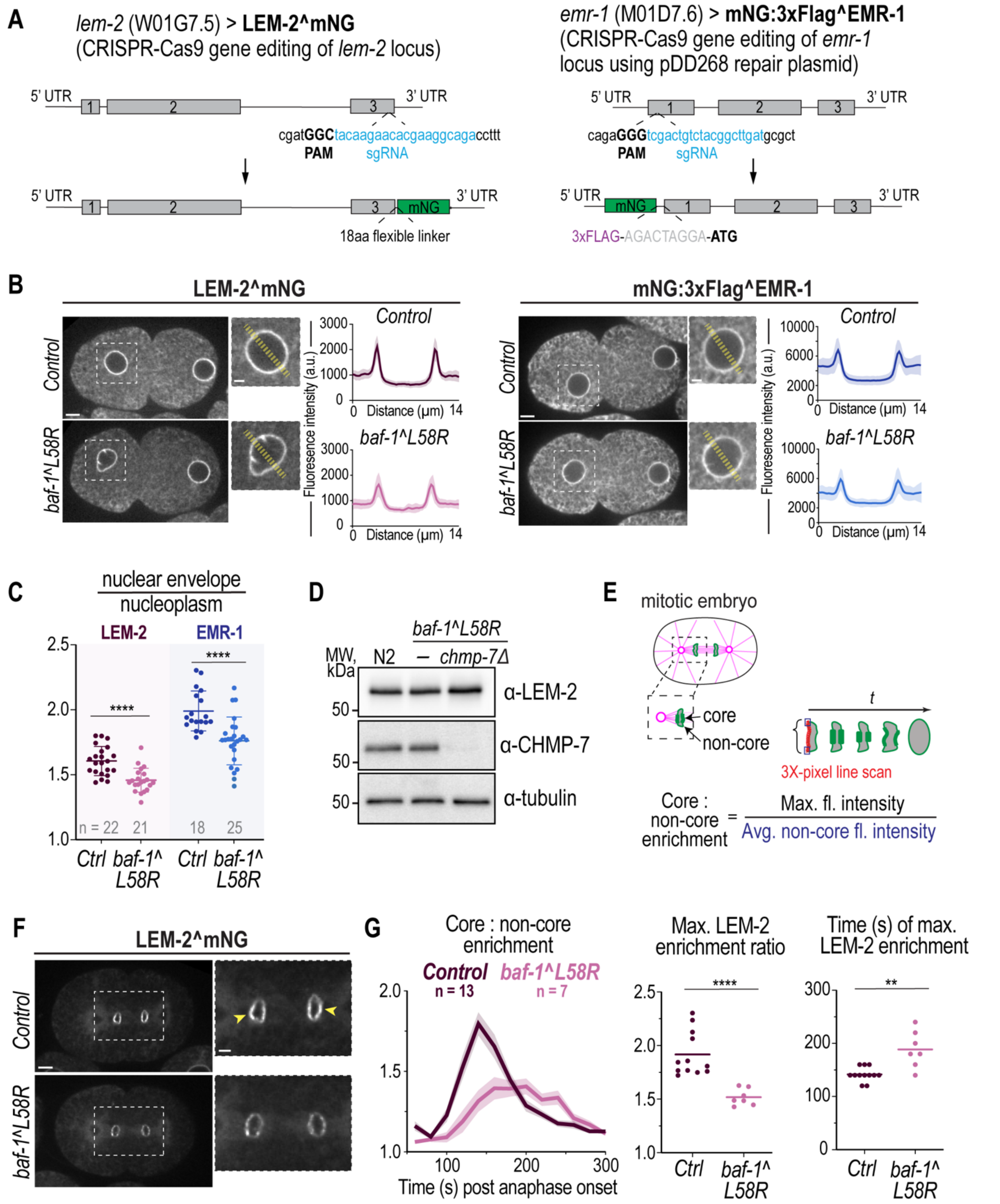

Fig. S3. Generation of endogenous mNG tagged strains and analysis of dynamics and protein levels of EMR-1 and LEM-2 in *baf-1(L58R)* mutant strain, related to Figure 3 (A) Schematic

Journal of Cell Science • Supplementary information

representations of endogenous gene loci of *emr-1* and *lem-1* with CRISPR guides used to insert mNG-tag. (B) Left, representative confocal images of LEM-2^mNG and mNG^EMR-1 at oocyte- and sperm-derived pronuclei at 200 seconds prior to pseudocleavage (PC) regression. Right, line scan analysis (average + SD) of LEM-2^mNG and mNG^EMR-1 at oocyte pronucleus at -200 sec relative to PC regression. n = # of embryos. (C) Plot (average ± SD + replicates) of NE:nucleoplasmic ratios of indicated proteins at oocyte pronucleus in indicated conditions. Statistical significance determined by unpaired Student's t-test (****=$p<0.0001$). n = # of embryos. (D) Representative immunoblot of whole worm lysates incubated with indicated antibodies in indicated strains. N = 3 independent experiments. (E) Schematic representations of core and non-core regions on reforming NE in mitotic *C. elegans* embryo and line scan analysis of LEM-2^mNG protein dynamics shown in (G). (F) Spinning disk confocal images of endogenous LEM-2^mNG enrichment at 140 seconds post anaphase onset. Yellow arrows, core enrichment. Scale bars, 5 µm. (G) Left, Plot (average + SEM) of ratio of maximum fluorescence intensity and non-core fluorescence signal measured in 20 second intervals from anaphase onset (t = 0) in indicated conditions, Middle, Plot (average + replicates) of maximum normalized LEM-2^mNG fluorescence signal from plot on left in indicated conditions. Statistical significance determined by Mann-Whitney test (****, $p < 0.0001$). Right, Plot (average + replicates) representing time of maximum fluorescence signal in indicated conditions. Statistical significance determined by Mann-Whitney test (**, $p = 0.002$). n = # of embryos.

Journal of Cell Science • Supplementary information

Barger et al. Figure S4

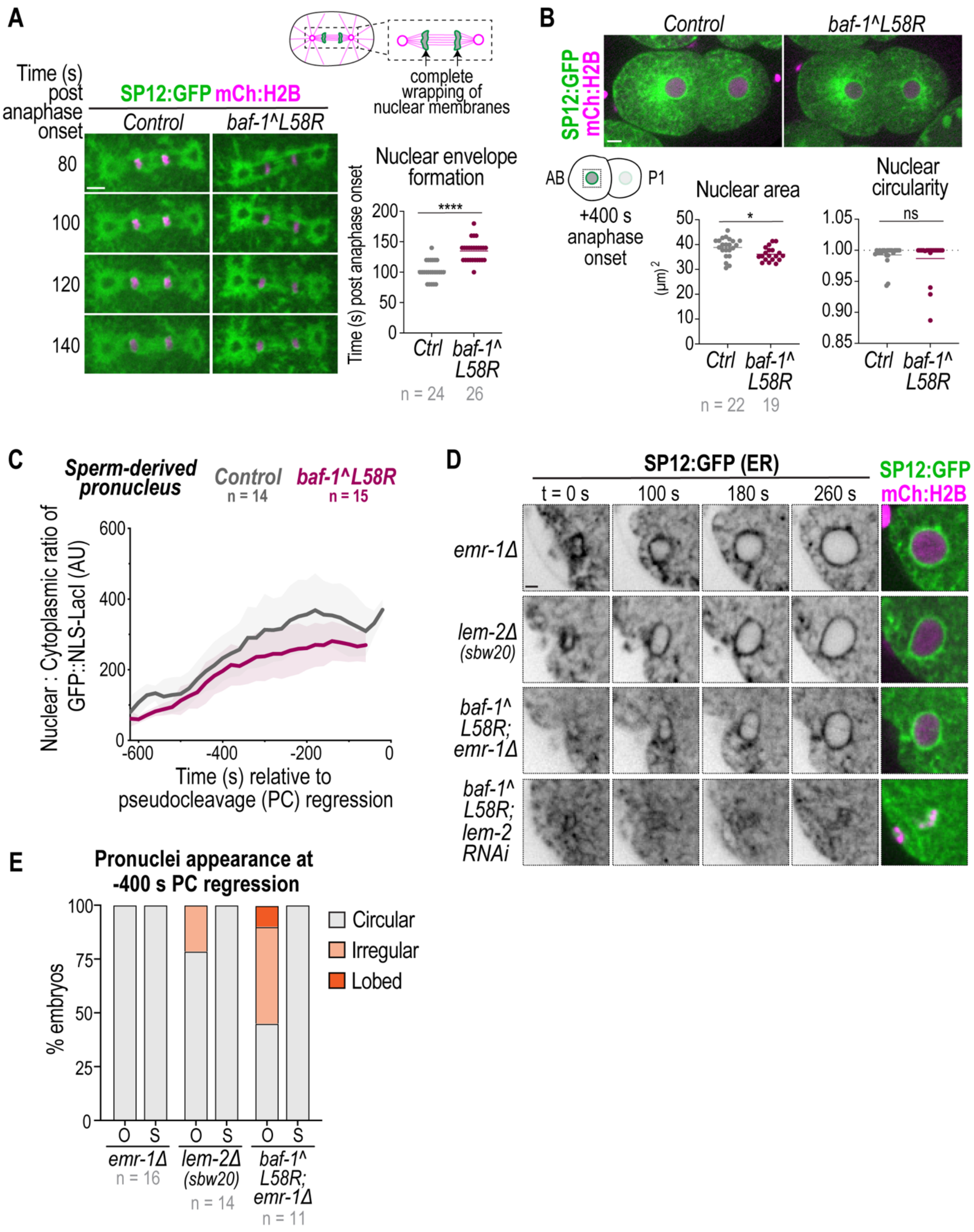

Journal of Cell Science • Supplementary information

Fig. S4. Analysis of pronuclear assembly in *emr-1Δ* and *lem-2Δ* mutants, related to Figure 4 (A) Left, representative confocal images from time-lapse series of SP12:GFP (ER marker) and mCh:Histone 2B (DNA marker) in mitotic embryos in indicated conditions. Time in seconds relative to anaphase onset. Right, plot (average + replicates) of time of complete wrapping of ER marker (SP12:GFP) around segregated chromosomes in indicated conditions. Statistical significance determined by unpaired Student's t-test (****=p<0.0001). n = # of embryos. (B) Above, spinning disk confocal images of indicated markers at 400 seconds post anaphase onset in indicated conditions. Below, schematic representation of 2-cell embryo and plots (average + replicates) of AB cell nuclear area and circularity at 400 seconds post anaphase onset in indicated conditions. Statistical significance determined by unpaired Student's t-test (*=p=0.0149, ns, not significant). n = # of embryos. Scale bar, 5 µm. (C) Plot (average ± SD) of normalized nuclear GFP::NLS-LacI fluorescence in sperm-derived pronucleus for indicated conditions. Time in seconds relative to pseudocleavage (PC) regression. Statistical significance at time points -500, -400 and -300 seconds PC regression was determined by unpaired Student's t-test (ns, not significant for all time points). n = # of embryos. (D) Spinning disk confocal images from time lapse series of indicated markers during oocyte-derived pronuclear formation in indicated conditions. Scale bar, 2 µm. (E) Plot representing percentage of oocyte-derived (O) and sperm-derived (S) pronuclei categorized as circular, irregular, or lobed at -400 seconds relative to pseudocleavage (PC) regression in indicated conditions. n = # of embryos.

Barger et al. Figure S5

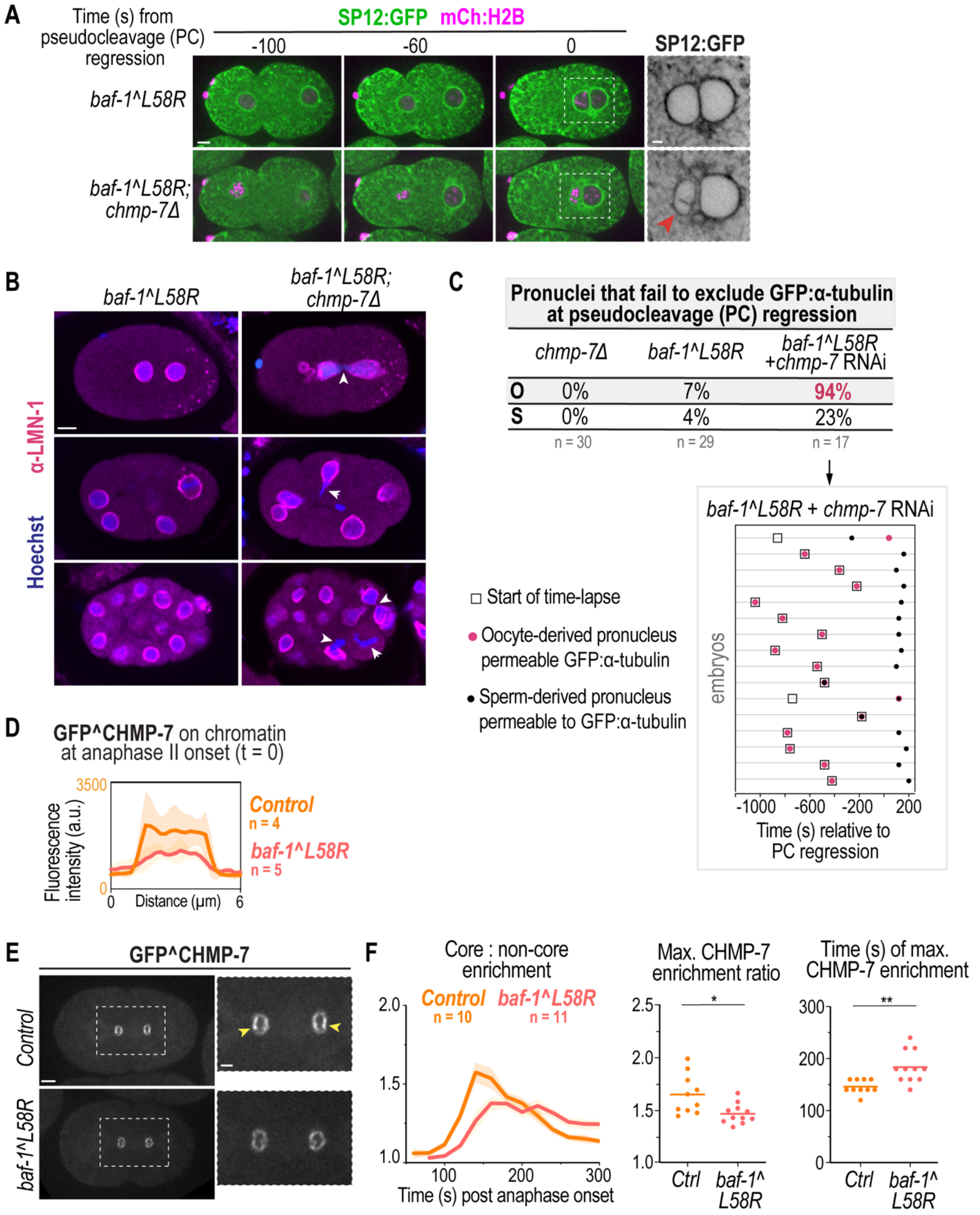

Journal of Cell Science • Supplementary information

Fig. S5. Analysis of loss of CHMP-7 and mNG^CHMP7 dynamics in meiosis and mitosis in *baf- 1-L58R* mutant embryos, related to Figure 5 (A) Spinning disk confocal images from time lapse series of indicated markers in indicated conditions. Time in seconds relative to pseudocleavage (PC) regression. Scale bar, 5 µm, zoom inset image scale bar, 2 µm. (B) Confocal images of fixed 2-,8-,16+ -cell stage *C. elegans* embryos immunostained with indicated markers (LMN-1 = lamin) in indicated conditions. White arrows point to chromatin bridges and impaired nuclear assembly. Scale bar, 10 µm (C) Above, table showing percentage of oocyte-derived (O) and sperm-derived (S) pronuclei permeable to GFP:α-tubulin at pseudocleavage (PC) regression in indicated conditions. n = # of embryos. Below, Plot representing time points for individual embryos containing nuclear GFP:α-tubulin in oocyte- (red circles) or sperm-derived pronuclei (black circles) relative to start of time-lapse movie (square). Time in seconds relative to pseudocleavage (PC) regression. (D) Background-corrected line scan analysis (average ± SD) of GFP^CHMP-7 on the nuclear rim at anaphase II onset (white arrows in Figure 5F) in indicated conditions. n = # of embryos. (E) Spinning disk confocal images of GFP^CHMP-7 at mitotic nuclear formation at 140 seconds post anaphase onset. Yellow arrows point to GFP^CHMP-7 core domain enrichment. Scale bars, 5 µm. (F) Left, plot (average + SEM) of ratio of maximum fluorescence intensity and non-core fluorescence signal measured in 20 second intervals from anaphase onset (t = 0) in indicated conditions, Middle, Plot (average + replicates) of maximum normalized GFP^CHMP-7 fluorescence signal from plot on left in indicated conditions. Statistical significance determined by unpaired Student's t-test (*, p = 0.0105). Right, plot (average + replicates) representing time of maximum fluorescence signal in indicated conditions. n = # of embryos. Statistical significance determined by unpaired Student's t-test (**, p = 0.0021). n = # of embryos.

Journal of Cell Science • Supplementary information

Barger et al. Figure S6

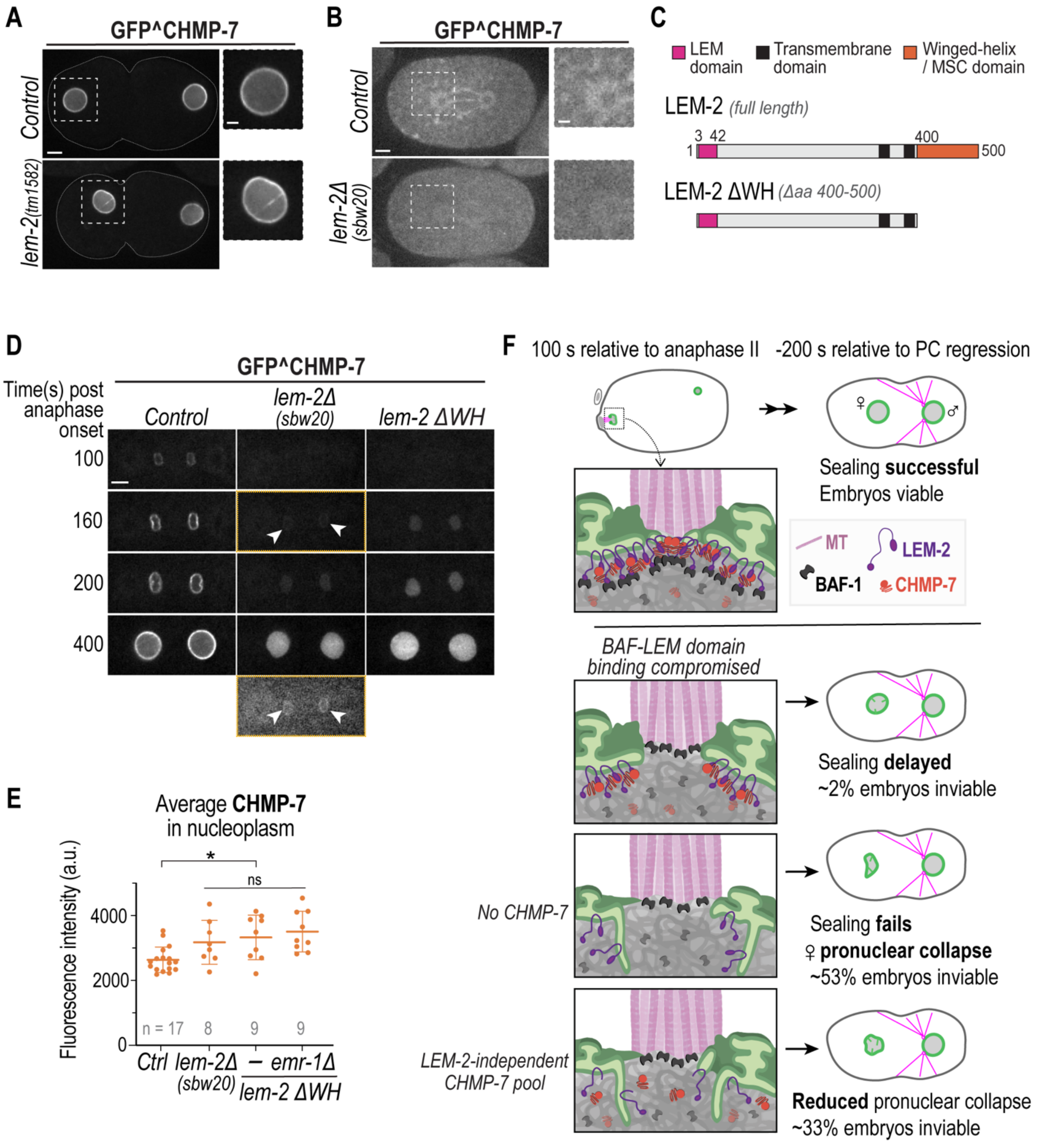

Fig. S6. CHMP-7 localization and dependence on LEM-2, related to Figure 6 (A) Spinning disk confocal images of GFP^CHMP-7 in embryos at -200 seconds relative to pseudocleavage (PC) regression in indicated conditions. White box and zoom inset: oocyte-derived pronucleus. Scale bars, 5 µm, zoom scale bars, 2 µm. (B) Spinning disk confocal images of endogenous GFP^CHMP-7 in control and *lem-2Δ* embryos at anaphase onset in mitotic embryos. Zoom inset of spindle pole. Scale bar, 5 µm, zoom scale

Journal of Cell Science • Supplementary information

bar, 2 μm. (C) Schematic representation of LEM-2 protein domain structure illustrating separation-of-function strain *lem-2ΔWH* (D) Spinning disk confocal images from time series of GFP^CHMP-7 in indicated conditions. Time in seconds relative to anaphase onset. Scale bar, 5 μm. White arrowheads point to faint fluorescence signal of GFP^CHMP-7 prior to nuclear accumulation. Yellow outlined panel reproduced below with brightness/contrast adjusted. (E) Plot (average ± SD + replicates) of nucleoplasmic GFP^CHMP-7 levels in oocyte-derived pronuclei -200 seconds relative to pseudocleavage (PC) regression. Statistical significance determined by one-way ANOVA and Tukey's post-hoc test (* = p = 0.0265, ns, not significant). n = # of embryos. (F) Schematic representation of oocyte-derived pronuclear sealing in different mutant backgrounds analyzed in this study (left). Right, zoom inset of sealing plaque adjacent to spindle microtubules. LEM domain proteins (only LEM-2 is shown for simplicity) attach incoming ER membranes to BAF to narrow the nuclear envelope hole. LEM-2/CHMP- 7 stabilize the nuclear envelope hole and remodel abnormal membranes, while a LEM-2 independent pool of CHMP-7 promotes nuclear stability through an unknown mechanism.

Journal of Cell Science • Supplementary information

Table S1. *C. elegans* strains used in this study

C. elegans strain name	Genotype	Source
N2	(Bristol): Wild-type (ancestral)	Caenorhabditis Genetics Center (CGC)
SBW69	unc-119 (ed3) III; sbwSi6 [mex-5p::baf-1(re-encoded)::tbb-2]; cb-unc-119(+) I	This study
SBW130	unc-119 (ed3) III; sbwSi12 [mex-5p::baf-1(re-encoded)-G47E::tbb-2 3']; cb-unc-119(+) I	This study
SBW131	unc-119 (ed3) III; sbwSi13 [mex-5p::baf-1(re-encoded)-L58R::tbb-2 3' UTR)]; cb-unc-119(+) I	This study
SBW144	baf-1(syb2609) III	This study; Suny Biotech
SBW136	mNG::3XFlag::baf-1(sbw7) III	This study
SBW142	mNG::3XFlag::baf-1(sbw7) III; unc-119(ed3) III; ltIs37 [pAA64; pie-1p::mCherry::his-58; unc-119 (+)] IV	This study
SBW152	mNG::3XFlag::baf-1(sbw7) III; weIs21 [pJA138 (pie-1p::mCherry::tubulin::pie-1)]	This study
SBW198	mNG::3XFlag::baf-1(L58R) III. [+/-]	This study
SBW253	emr-1(gk119) I; mNG::3XFlag::baf-1(sbw7) III; unc-119(ed3) III; ltIs37 [pAA64; pie-1p::mCherry::his-58; unc-119 (+)] IV.	This study
VC237	emr-1(gk119) I	Caenorhabditis Genetics Center (CGC)
SBW448	lem-2 (sbw20) II	This study
TG4319	lem-3(tm3468) I	Caenorhabditis Genetics Center (CGC)
SBW174	emr-1(gk119) I; baf-1(syb2609) III	This study
SBW180	lem-3(tm3468) I; baf-1(syb2609) III	This study

Journal of Cell Science • Supplementary information

SBW47	unc-119(ed3) III; ltIs37 [pAA64; pie-1p::mCherry::his-58; unc-119 (+)] IV; ltIs75 [(pSK5) pie-1::GFP::TEV-STag::LacI + unc-119(+)].	(Audhya et al., 2007)
SBW170	baf-1(syb2609) III ; unc-119(ed3) III; ltIs37 [pAA64; pie-1p::mCherry::his-58; unc-119 (+)] IV; ltIs75 [(pSK5) pie-1::GFP::TEV-STag::LacI + unc-119(+)].	This study
SBW181	emr-1(gk119) I; unc-119(ed3) III; ltIs37 [pAA64; pie-1p::mCherry::his-58; unc-119 (+)] IV; ltIs75 [(pSK5) pie-1::GFP::TEV-STag::LacI + unc-119(+)].	This study
SBW457	lem-2 (sbw20) I ; unc-119(ed3) III; ltIs37 [pAA64; pie-1p::mCherry::his-58; unc-119 (+)] IV; ltIs75 [(pSK5) pie-1::GFP::TEV-STag::LacI + unc-119 (+)].	This study
OD270	unc-119(ed3) III; ojls23 [SP12::GFP; unc-119(+)]; ltIs37 [pAA64; pie-1p::mCherry::his-58; unc-119 (+)]	(Bahmanyar et al., 2014)
SBW173	baf-1(syb2609) III; ojls23 [SP12::GFP; unc-119(+)]; ltIs37 [pAA64; pie-1p::mCherry::his-58; unc-119 (+)] IV	This study
SBW199	emr-1(gk119) I ; ojls23 [SP12::GFP; unc-119(+)]; ltIs37 [pAA64; pie-1p::mCherry::his-58; unc-119 (+)] IV	This study
SBW543	lem-2 (sbw20) II ; ojls23 [SP12::GFP; unc-119(+)]; ltIs37 [pAA64; pie-1p::mCherry::his-58; unc-119 (+)] IV	This study
SBW32	ltIs24 [pAZ132; pie-1p::GFP::tba-2; unc-119 (+)]; ltIs37 [pAA64; pie-1p::mCherry::his-58; unc-119 (+)] IV	(Penfield et al., 2018)
SBW171	baf-1(syb2609) III; ltIs24 [pAZ132; pie-1p::GFP::tba-2; unc-119 (+)]; ltIs37 [pAA64; pie-1p::mCherry::his-58; unc-119 (+)] IV	This study
SBW480	emr-1(gk119) I; ltIs24 [pAZ132; pie-1p::GFP::tba-2; unc-119 (+)]; ltIs37 [pAA64; pie-1p::mCherry::his-58; unc-119 (+)] IV	This study
SBW455	lem-2 (sbw20) II; ltIs24 [pAZ132; pie-1p::GFP::tba-2; unc-119 (+)]; ltIs37 [pAA64; pie-1p::mCherry::his-58; unc-119 (+)] IV	This study
SBW146	lem-2::mNG (sbw5) II; unc-119(ed3) III; ltIs37 [pAA64; pie-1p::mCherry::his-58; unc-119 (+)] IV.	This study
SBW206	lem-2::mNG (sbw5) II; baf-1(syb2609) III; unc-119(ed3) III; ltIs37 [pAA64; pie-1p::mCherry::his-58; unc-119 (+)] IV	This study

Journal of Cell Science • Supplementary information

SBW414	gfp::chmp-7 II; unc-119(ed3) III; ltIs37 [pAA64 pie-1p::mCherry::his-58; unc-119 (+)] IV	This study
SBW428	gfp::chmp-7 II; baf-1(syb2609) III ; unc-119(ed3) III; ltIs37 [pAA64; pie-1p::mCherry::his-58; unc-119 (+)] IV	This study
SBW312	mNG::3xFlag::emr-1 (sbw16) I. outcrossed 4X; unc-119(ed3) III; ltIs37 [pAA64; pie-1p::mCherry::his-58; unc-119 (+)] IV	This study
SBW350	mNG::3xFlag::emr-1 (sbw16) I ; baf-1(syb2609) III ; unc-119(ed3) III; ltIs37 [pAA64; pie-1p::mCherry::his-58; unc-119 (+)] IV.	This study
SBW161	chmp-7(hz12) II; unc-119(ed3) III; ojls23[SP12::GFP unc-119(+)]; unc-119(ed3) III; ltIs37 [pAA64; pie-1p::mCherry::his-58; unc-119 (+)] IV	This study
SBW196	chmp-7(hz12) II; baf-1(syb2609) III; unc-119(ed3) III; ojls23 [SP12::GFP; unc-119(+); ltIs37 [pAA64; pie-1p::mCherry::his-58; unc-119 (+)] IV	This study
SBW63	chmp-7(hz12) II; ltIs24 [pAZ132; pie-1p::GFP::tba-2; unc-119 (+)]; ltIs37 [pAA64; pie-1p::mCherry::his-58; unc-119 (+)] IV	(Penfield et al., 2020)
SBW526	emr-1(gk119) I; gfp::chmp-7 II; unc-119(ed3) III; ltIs37 [pAA64; pie-1p::mCherry::his-58; unc-119 (+)] IV	This study
SBW416	gfp::chmp-7 II; lem-2(tm1582) unc-119(ed3) III; ltIs37 [pAA64 pie-1p::mCherry::his-58; unc-119 (+)] IV	This study
SBW458	lem-2 (sbw20) II; gfp::chmp-7 II; unc-119(ed3) III; ltIs37 [pAA64; pie-1p::mCherry::his-58; unc-119 (+)] IV	This study
SBW419	gfp::chmp-7 II; lem-2 (syb4718) II; unc-119(ed3) III; ltIs37 [pAA64; pie-1p::mCherry::his-58; unc-119 (+)] IV	This study
SBW547	emr-1(gk119) I; gfp::chmp-7 II; lem-2 (syb4718) II; unc-119(ed3) III; ltIs37 [pAA64; pie-1p::mCherry::his-58; unc-119 (+)] IV	This study
SBW332	lem-2 (syb4718) II; ojls23 [SP12::GFP; unc-119(+)]; ltIs37 [pAA64; pie-1p::mCherry::his-58; unc-119 (+)] IV	This study
SBW536	lem-2 (syb4718) II; baf-1(syb2609) III; ojls23 [SP12::GFP; unc-119(+)]; ltIs37 [pAA64; pie-1p::mCherry::his-58; unc-119 (+)] IV	This study

Journal of Cell Science • Supplementary information

MSN772	chmp-7(hz12) II	(Penfield et al., 2020)
SBW168	chmp-7(hz12) II; baf-1(syb2609) III	This study
SBW454	emr-1(gk119) I; chmp-7(hz12) II	This study
SBW450	lem-2 (sbw20) II; chmp-7(hz12) II	This study
SBW319	lem-2 (syb4718) II	This study; Suny Biotech
SBW348	lem-2 (syb4718) II; baf-1(syb2609) III	This study

Journal of Cell Science • Supplementary information

Table S2. Oligonucleotides used in this study.

Purpose	Primers
pCFJ151-baf-1-G47E mutagenesis-Forward	GCCTACGTCTTGTTCGAACAGTATCTCCTGCTT
pCFJ151-baf-1-G47E mutagenesis-Reverse	AAGCAGGAGATACTGTTCGAACAAGACGTAGGC
pCFJ151-baf-1-L58R mutagenesis-Forward	AAAAAGGATGAGGATCGTTTCATCGAGTGGCTG
pCFJ151-baf-1-L58R mutagenesis-Reverse	CAGCCACTCGATGAAACGATCCTCATCCTTTTT

**Oligonucleotides for dsRNA production**			
Gene	Oligonucleotide 1	Oligonucleotide 1	Template
B0464.7 (*baf-1*)	TAATACGACTCACTATAGGCAT CGTGAGTTCGTCGGAGA	TAATACGACTCACTATAG Ggtccaagaccacacgacaag	N2 genomic
Y55F3BR .8 (*lem-4*)	TAATACGACTCACTATAGGCCG AGCAATCAGAAGCCATG	TAATACGACTCACTATAG GTTGCATGGCTCATCATCT GC	N2 genomic
T24B8.2 (*chmp-7*)	TAATACGACTCACTATAGGTCG GTGAATGGAGAGATCGT	TAATACGACTCACTATAG GGTTCTGAGCACGTCCTTT GT	N2 genomic
M01D7.6 *(emr-1)*	AATTAACCCTCACTAAAGGCGA ACTACGCGATAGCCTTA	TAATACGACTCACTATAG GCCCAAGAATCCTCCTTT GTT	N2 genomic
W01G7.5 *(lem-2)*	TAATACGACTCACTATAGGAGA AAATGTCGGATGCAGAG	TAATACGACTCACTATAG GTTGTTAGGCGTCGAAGA AAC	N2 genomic

Journal of Cell Science • Supplementary information

Table S3. Recombinant DNA used in this study.

Identifier	Recombinant DNA	Source
	baf-1 re-encoded	Genescript
pSB353	pCFJ151-baf-1-WT reencoded	This study
pSB419	pCFJ151-baf-1-G47E reencoded	This study
pSB420	pCFJ151-baf-1-L58R reencoded	This study
	pDD122 (CRISPR-Cas9)	(Hastie et al., 2019)
	pDD268 (mNG^SEC^3xFlag vector with ccdB markers for cloning homology arms)	Addgene (132523)
	LL-mNG (mNG^SEC vector without ccdB markers)	(Hastie et al., 2019)
pSB457	pDD122-lem-2	This study
pSB462	lem-2-LL-mNG	This study
pSB495	pDD122-baf-1	This study
pSB496	pDD268-baf-1	This study
pSB514	pDD122-emr-1	This study
pSB651	pDD268-emr-1	This study

Journal of Cell Science • Supplementary information

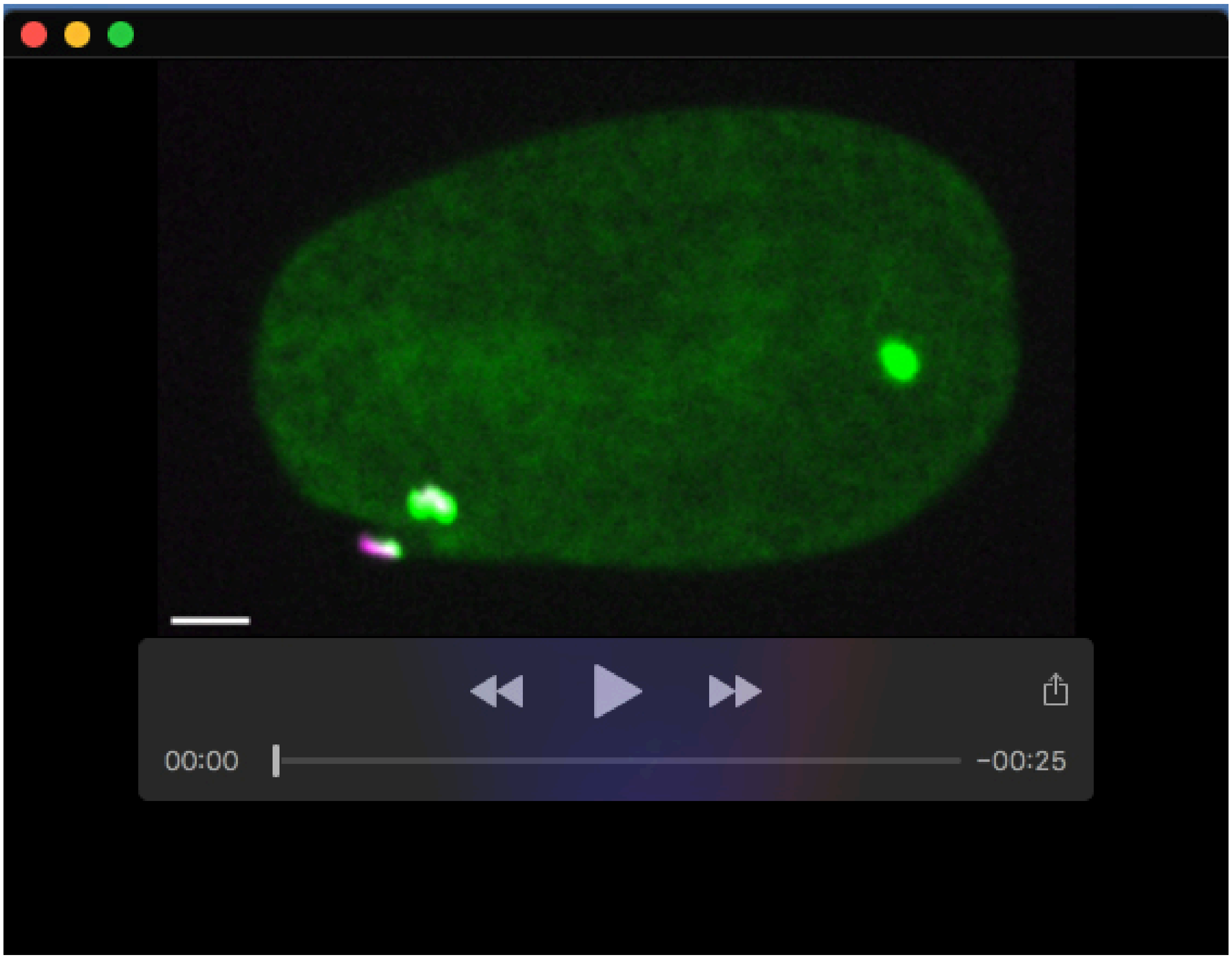

Movie 1. BAF-1 dynamics at oocyte- and sperm-derived pronuclei during pronuclear formation, related to Figure 1. Spinning disk confocal fluorescence time series of mNG^BAF-1 (green) and mCherry:Histone2B (magenta) in fertilized oocyte. Time lapse shows mNG^BAF-1 enrichment on chromatin transitioning to nuclear membranes during oocyte- (left) and sperm- (right) derived pronuclear formation. Images were acquired every 20 s – playback rate is 80X real time. Scale bar, 5 µm.

Journal of Cell Science • Supplementary information

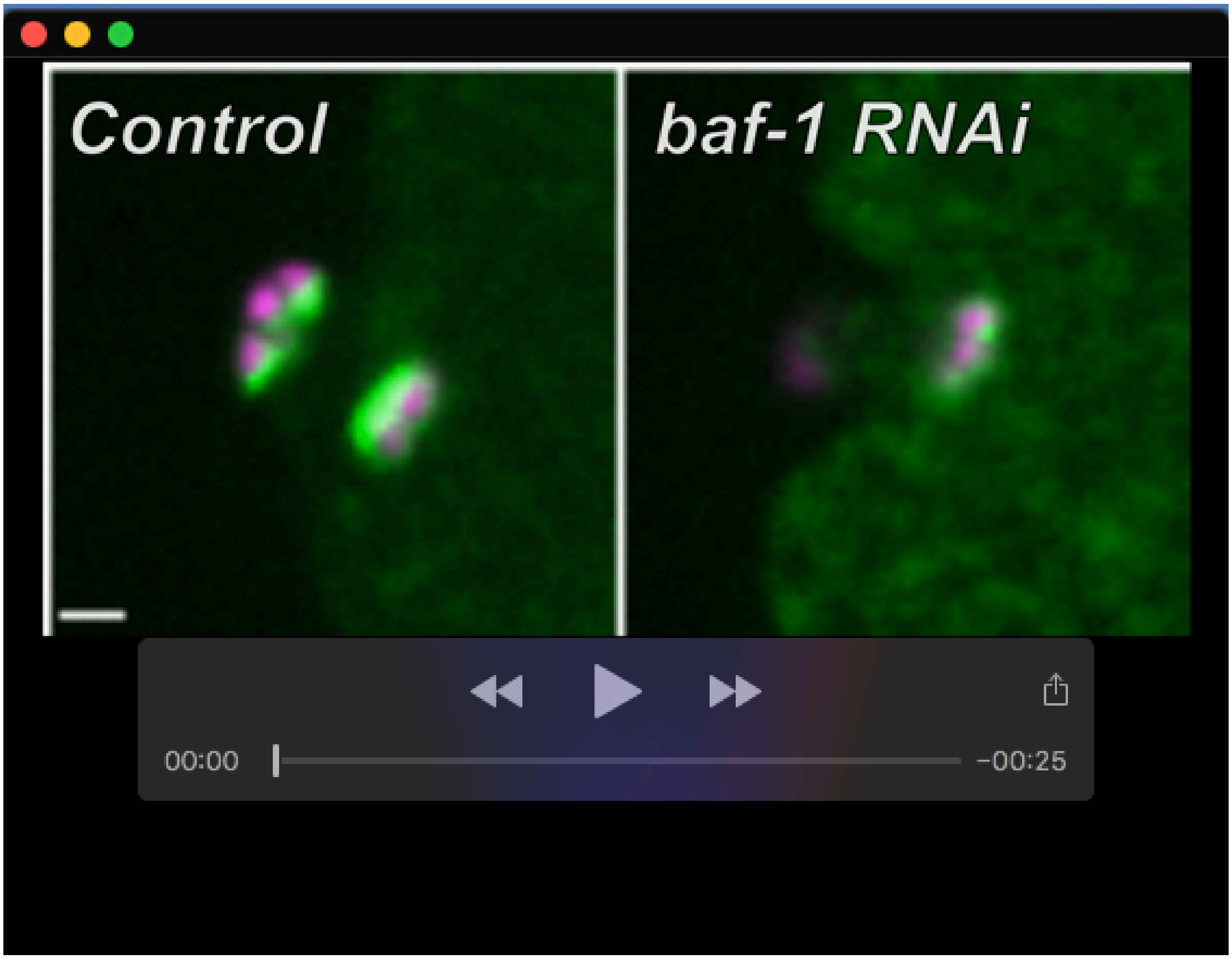

Movie 2. BAF-1 is required for LEM-2 enrichment at the sealing plaque following meiosis II, related to Figure 1. Spinning disk confocal fluorescence time series of endogenously tagged LEM-2^mNG (green) and mCherry:Histone2B (magenta) in fertilized oocytes. Time lapse shows oocyte-derived pronuclear formation, starting at anaphase II onset, as chromatin moves away from the cortex, and ending with a sealed pronucleus in indicated conditions. LEM-2^mNG does not accumulate at a discrete focus in the reforming oocyte-derived pronucleus, which undergoes assembly failure. Images were acquired every 20 s – playback rate is 80X real time. Scale bar, 2 µm.

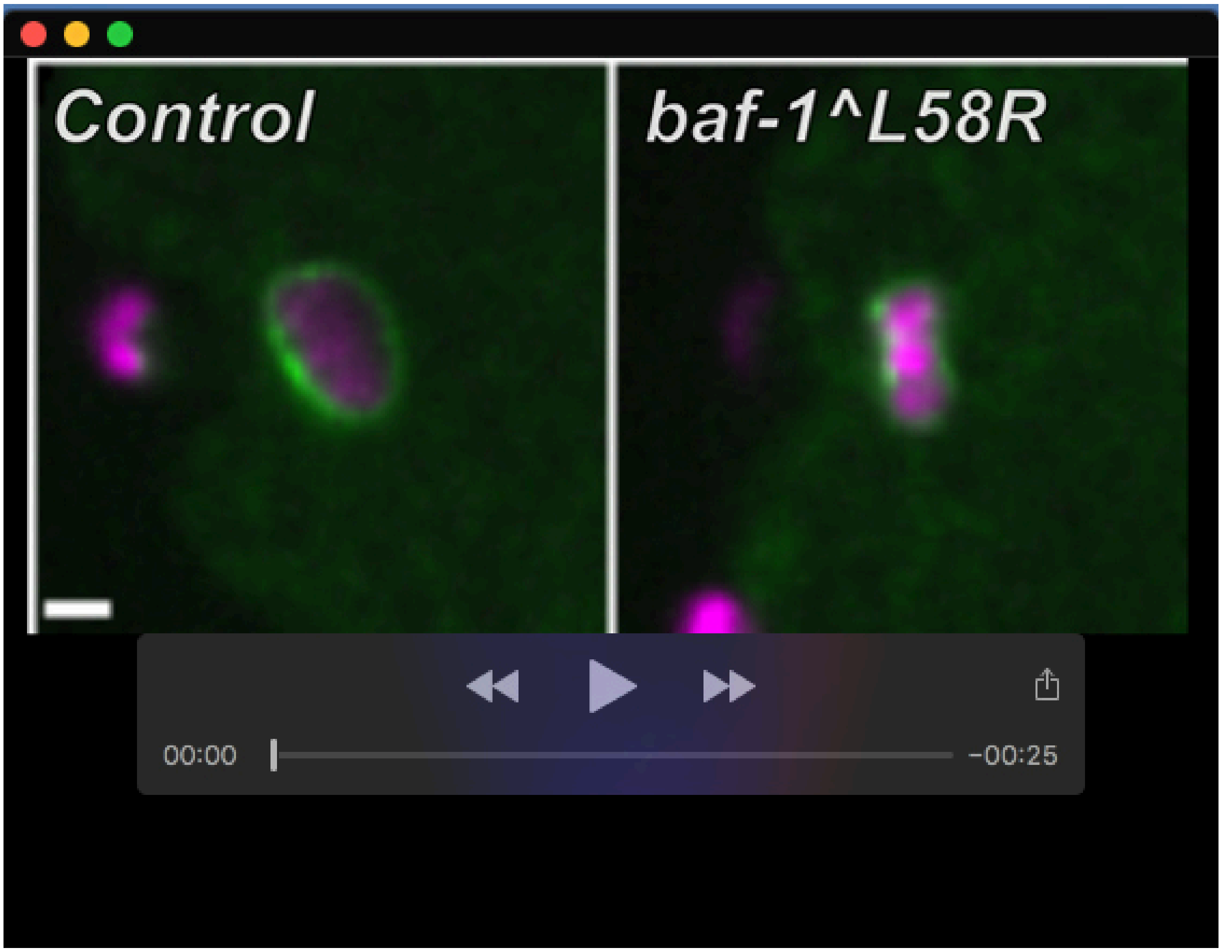

Movie 3. Reduced and delayed LEM-2^mNG enrichment at the sealing plaque in *baf-1^L58R* embryos, related to Figure 3. Spinning disk confocal fluorescence images of LEM-2^mNG (green) and mCherry:Histone2B (magenta) in fertilized oocytes in indicated conditions. Time lapse shows oocyte-derived pronuclear formation, starting at anaphase II onset. Images were acquired every 20 s – playback rate is 80X real time. Scale bar, 2 µm.

Journal of Cell Science • Supplementary information

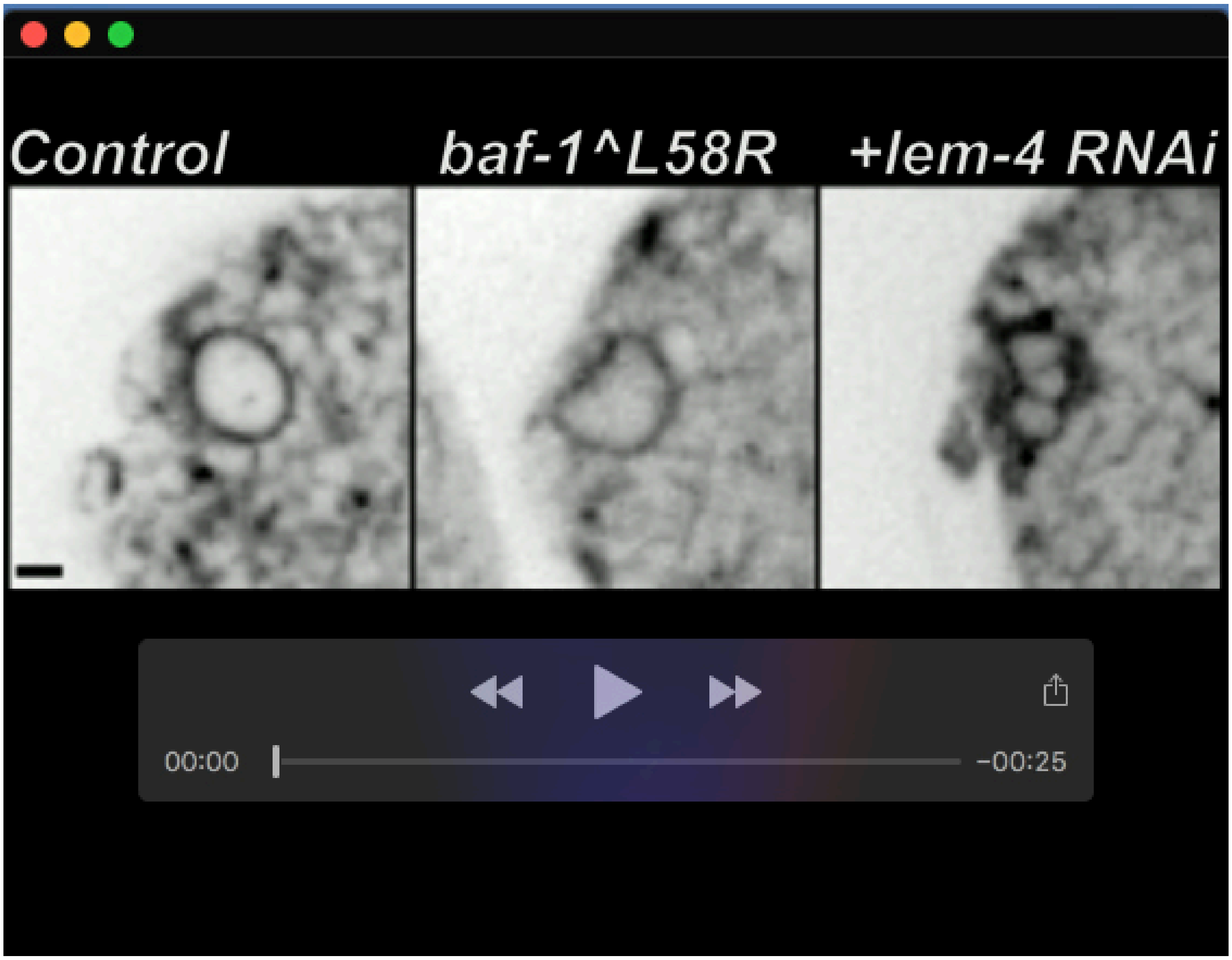

Movie 4. Oocyte pronuclear formation in control, *baf-1^L58R*, and *baf-1^L58R* + *lem-4* RNAi embryos, related to Figure 4. Spinning disk confocal fluorescence time series of embryos expressing SP12:GFP (ER in grey, inverted) in indicated conditions. Images were acquired every 20 s – playback rate is 80X real time. Scale bar, 2 µm.

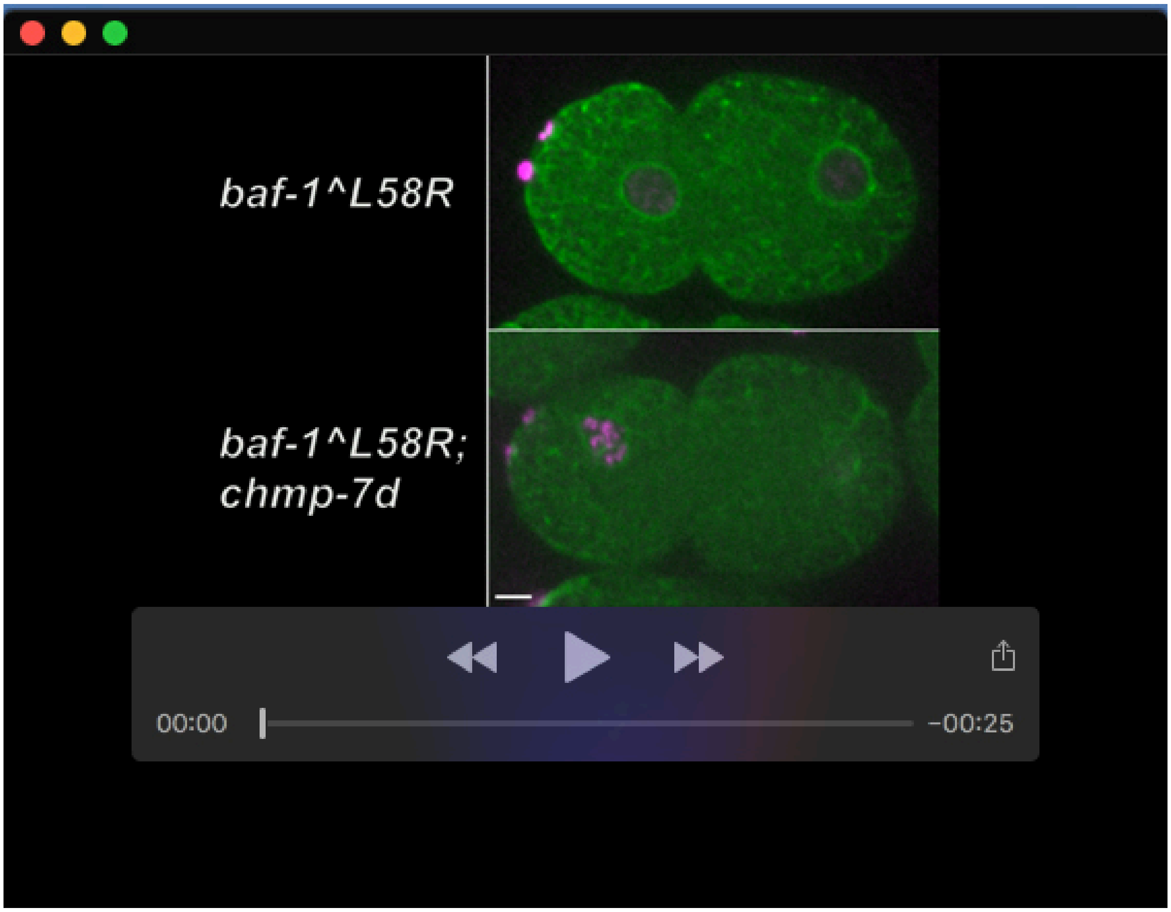

Movie 5. Oocyte pronuclear collapse occurs in *chmp-7*Δ; *baf-1^L58R* embryos, related to Figure 5. Spinning disk confocal fluorescence time series of SP12:GFP (ER, green) and mCherry:Histone2B (magenta) marking oocyte-derived pronuclei in indicated conditions. Time lapse shows migration of oocyte- and sperm-derived pronuclei ending in pronuclear meeting approximately at PC regression. Images were acquired every 20 s – playback rate is 80X real time. Scale bar, 5 µm.

Journal of Cell Science • Supplementary information

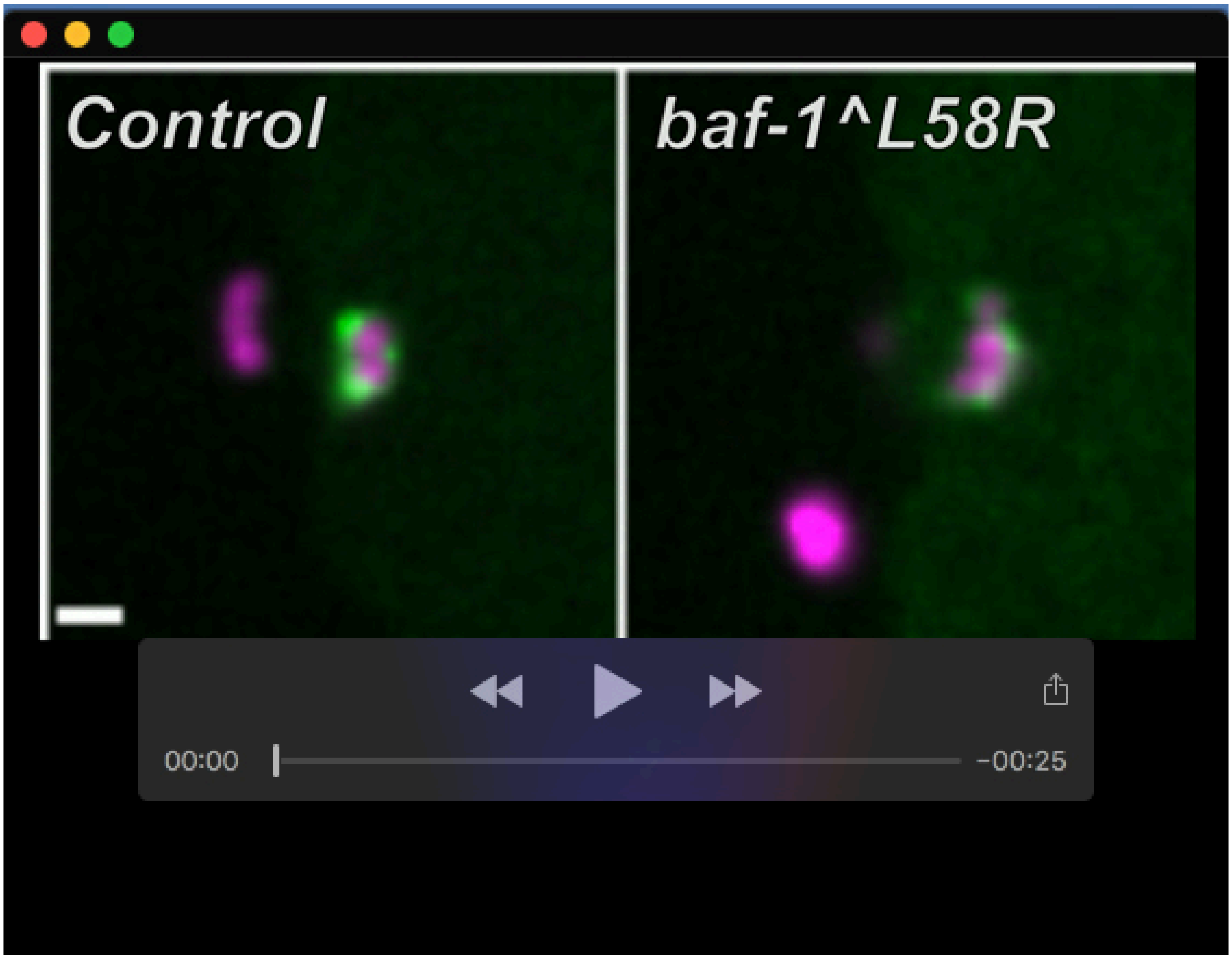

Movie 6. Reduced and delayed CHMP-7 enrichment at the sealing plaque in *baf-1^L58R* embryos, related to Figure 5. Spinning disk confocal fluorescence time series of GFP^CHMP-7 (green) and mCherry:Histone2B (magenta) after anaphase II onset in fertilized oocytes in indicated conditions. Time lapse shows oocyte-derived pronuclear formation, starting at anaphase II onset as chromatin moves away from the cortex and ending with a formed pronucleus in indicated conditions. Images were acquired every 20 s – playback rate is 80X real time. Scale bar, 2 µm.

Journal of Cell Science • Supplementary information

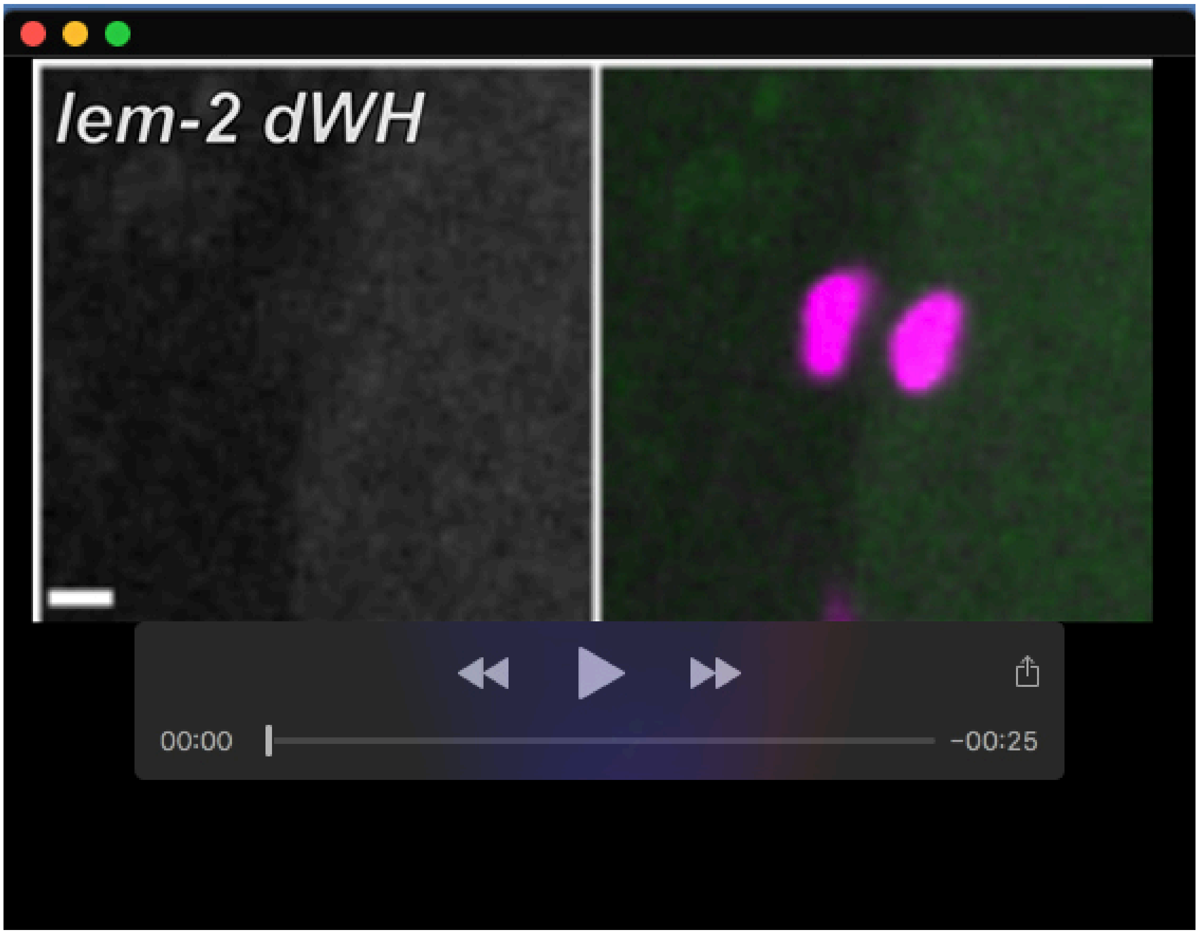

Movie 7. Reliance of GFP^CHMP-7 on the Winged Helix domain of LEM-2 to localize and enrich at the sealing plaque following meiosis II, related to Figure 6. Spinning disk confocal fluorescence time series of GFP^CHMP-7 (green) and mCherry:Histone2B (magenta) after anaphase II onset in fertilized oocytes in indicated conditions. Left, gray scale images of GFP^CHMP-7, Right, merged. Time lapse shows oocyte-derived pronuclear formation, starting at anaphase II onset as chromatin moves away from the cortex and ending with a formed pronucleus in *lem-2* Δ*WH* mutant. Left, GFP^CHMP-7 (grey), Right, merged. Images were acquired every 20 s – playback rate is 80X real time. Scale bar, 2 µm.

References

Audhya, A., Desai, A. and Oegema, K. (2007). A role for Rab5 in structuring the endoplasmic reticulum. *J. Cell Biol.* **178**, 43-56. doi:10.1083/jcb.200701139

Bahmanyar, S., Biggs, R., Schuh, A. L., Desai, A., Müller-Reichert, T., Audhya, A., Dixon, J. E. and Oegema, K. (2014). Spatial control of phospholipid flux restricts endoplasmic reticulum sheet formation to allow nuclear envelope breakdown. *Genes Dev.* **28**, 121-126. doi:10.1101/gad.230599.113

Journal of Cell Science • Supplementary information
