# Supplementary material for: Nuclear envelope assembly relies on CHMP-7 in the absence of BAF–LEM-mediated hole closure
Source: J Cell Sci. 2023 Nov 13;136(21):jcs261385. doi: 10.1242/jcs.261385 (PMC10668030; doi:10.1242/jcs.261385)
Supplement: Supplementary file 1 [file joces-136-261385_review_history.pdf]

Nuclear envelope assembly relies on CHMP-7 in the absence of BAF-LEM-mediated hole closure

Sarah R. Barger, Lauren Penfield and Shirin Bahmanyar
DOI: 10.1242/jcs.261385

Editor: Jennifer Lippincott-Schwartz

Review timeline

Original submission:	4 June 2023
Editorial decision:	22 August 2023
First revision received:	20 September 2023
Accepted:	21 September 2023

Original submission

First decision letter

MS ID#: JOCES/2023/261385

MS TITLE: Nuclear envelope assembly relies on CHMP-7 in the absence of BAF-LEM-mediated hole closure

AUTHORS: Sarah R Barger, Lauren Penfield, and Shirin Bahmanyar
ARTICLE TYPE: Research Article

We have now reached a decision on the above manuscript.

To see the reviewers' reports and a copy of this decision letter, please go to: https://submit-jcs.biologists.org and click on the 'Manuscripts with Decisions' queue in the Author Area. (Corresponding author only has access to reviews.)

As you will see, the reviewers gave favourable reports but raised some critical points that will require amendments to your manuscript. I hope that you will be able to carry these out because I would like to be able to accept your paper, depending on further comments from reviewers.

Please ensure that you clearly highlight all changes made in the revised manuscript. Please avoid using 'Tracked changes' in Word files as these are lost in PDF conversion.

I should be grateful if you would also provide a point-by-point response detailing how you have dealt with the points raised by the reviewers in the 'Response to Reviewers' box. Please attend to all of the reviewers' comments. If you do not agree with any of their criticisms or suggestions please explain clearly why this is so.

Reviewer 1

Advance summary and potential significance to field

This is a very well done and written manuscript on how the nuclear envelope seals after anaphase II of meiosis. It focuses nicely finding new mechanisms for how BAF, LEM proteins, and CHIMP7 function to form the new nuclear envelope in the presence of microtubule bundles. This is an important study that sheds new information on nuclear assembly. Interestingly, it used separation

© 2023. Published by The Company of Biologists under the terms of the Creative Commons Attribution License (https://creativecommons.org/licenses/by/4.0/).

of function alleles in BAF-1 to identify slightly different roles for the LEM proteins EMR-1 and LEM-2, which were previously thought to function redundantly. It found a number of new conclusions: That BAF binding to LEM is not essential; That LEM-2 is more important than previously reported and that it plays a role independent of EMR-1. I particularly liked how it contrasted the roles of these proteins in the female pronucleus which has a bundle of microtubules, and the male pronucleus which forms the NE at the same time in the absence of a MT bundle. Very cool. The figures are very well done and easy to follow. Given the maturity of the written manuscript and the figures, I suspect this has already been through the review process. I therefore have very few comments. It will be of interest to a broad group of cell biologists, including those interested in the nuclear envelope, micronuclei, DNA-membrane interactions, and early development including meiosis.

Comments for the author

No major revisions required!

Minor points:

1. I appreciate generation of the new lem-2 allele and setting the record straight on the tm1582 allele. Add allele names to the experiment. What is the new allele name? It should be near line 230. Also, add the allele name to figure 2g-h, is that the new allele? It is not clear. Also add the allele names to lines 354-355.
2. In Figure 3B and S3G, the colors of the two lines are too close to each other. Please pick a color pair with more contrast.

<u>Reviewer 2</u>

Advance summary and potential significance to field

Barger and coworkers report that the interaction of DNA-binding protein Barrier-to-autointegration factor BAF with LEM domain proteins is required for efficient and rapid closure of nuclear membranes following segregation of chromosomes in meiosis II in C. elegans embryos. Previous investigations have demonstrated that the two C. elegans LEM domains EMR-1 (emerin) and LEM-2 (LEMD2) have both redundant and unique functions. This is also observed in this study. By performing high-resolution life microscopy on strains expressing endogenously tagged BAF-1, EMR-1, LEM-2 and CHMP-7, the authors demonstrate that all four proteins accumulate on the surface of the assembling oocyte-derived pronucleus at the site where final membrane closure takes place (“sealing plaque”).
Depletion of BAF-1 by RNAi prevents accumulation of EMR-1 and LEM-2 and leads to the formation of highly aberrant pronuclei, which is in accordance with previous reports. Interestingly, a single point mutation in BAF-1 (L58R) that reduces the interaction with LEM domains has only a minor effect on fertility but leads to complete sterility in lem-2(0) mutants (but not when combined with emr-1(0)).
The baf-1(L58R) mutation delays accumulation of EMR-1 and LEM-2 and the sealing plaque and, presumably in consequence, slower pronuclear envelope sealing and nuclear protein import. In the case of CHMP-7, accumulation in not delayed in baf-1(L58R) embryos but CHMP-7 distribution is irregular and pronuclear morphology is dramatically worsened in baf-1(L58R); chmp-7(RNAi) embryos. The effects on pronuclear morphology and pronuclear envelope sealing are stronger on the oocyte-derived pronucleus, which must seal a larger membrane gap after disassembly of meiotic microtubules compared to the sperm-derived pronucleus.
Finally, the authors show that the WH domain of LEM-2 is require for efficient recruitment of CHMP-7 to reforming (pro-)nuclear envelopes, but not for nuclear accumulation of CHMP-7 nor viability.
The study is of high quality: the data are convincing, and the text provides a detailed description and discussion of the findings in the context of previous experiments performed in C. elegans and other organisms. I find that the manuscript provides an important advance in understanding the complex interplay between nuclear envelope proteins in nuclear assembly. However, I suggest the authors consider the following points prior to publication:

© 2023. Published by The Company of Biologists under the terms of the Creative Commons Attribution License (https://creativecommons.org/licenses/by/4.0/).

Comments for the author

1) The abstract argues that the relevance of BAF-LEM binding in membrane closure during mitosis is unknown and says in the following sentence that membrane closure is studied in meiosis in this study. This seems confusing. Is the mechanism the same in the two types of division?
2) All observations on protein localisation are based on mNG-tagged strains. By exclusively using endogenously tagged strains, expression levels are likely to reflect those of untagged proteins. Nevertheless, the mNG tag might still affect protein behaviour. Indeed, while both mNG-FLAG-BAF-1(wt) and BAF-1(L58R) are viable, the mNG-FLAG-BAF-1(L58R) strain is not viable in homozygosity, indicating an impact of mNG. Validating the accumulation at sealing plaques (or the absence thereof in baf-1 mutants) with antibodies against untagged proteins would strengthen the manuscript.
3) Related to the potential impact of mNG on fusion protein behaviour, why was mNG inserted at the N-terminus of EMR-1 and at the C-terminus of LEM-2? The ratio of NE/ER signal of LEM-2-mNG seems higher than that of mNG-EMR-1 – could the position of the tag influence this observation? Other studies in which EMR-1 was tagged at the C-terminus seem to have higher NE/ER signal ratio. Note that in line 238 the order is reversed (EMR-1-mNG).
4) In lines 203-204, the authors argue that binding to LEM domain proteins affect localisation of BAF-1 to the ER. However, the quantification of fluorescence signal seems to be based on ER + cytoplasm, i.e. not only ER.
Moreover, because mNG might stabilise BAF-1(wt) and/or destabilise BAF-1(L58R) the authors should demonstrate that mNG-FLAG-BAF-1 and mNG-FLAG-BAF-1(L58R) are expressed at the same level.
5) The discovery that the lem-2(tm1582) allele encodes a protein lacking aa 23-283 rather than just a short 53 aa peptide as previously assumed is quite important. However, a more detailed side-by-side comparison of phenotypes of the tm1582 and the novel null allele should be provided.
6) Related to the previous point, why was the in-frame deletion allele tm3468 of lem-3 used rather than the stronger mn155 allele?
7) Some graphs include results of statistical tests while others do not (e.g. Fig S3, C vs G).
8) Are the differences in nuclear import of GFP-NLS-LacI in Figure 4C and S4C statistically significant?
9) Regarding the argument in lines 282-284: “Sterility resulting from loss of lem-2 in baf-1(L58R) worms prevented us from genetically testing the consequences resulting from loss of lem-2 in baf-1(L58R) embryos.” This could be investigated by RNAi against lem-2 as for emr-1 and lem-4 in Fig 4E.

Reviewer 3

Advance summary and potential significance to field

This is an extremely careful and rigorous cell biological and genetic analysis of a challenging stage in nuclear envelope (NE) assembly, using the best possible biological system (meiotic prophase-II in C. elegans embryos). This manuscript reports the mechanisms of NE membrane sealing in the ‘core’ region where spindle microtubules (MTs) create large holes that interfere with the enclosure of chromosomes by nuclear membranes, focusing on the intertwined roles of BAF-1 and two ‘redundant’ LEM-domain proteins (Emr-1/emerin and LEMD2), as well as Lem3. They made careful use of a BAF-1 mutation (L58R) defective in binding to LEM-domain proteins. The authors reveal a long-standing annotation mistake in the field about the null phenotype of LEMD2, which allowed them to discover that the winged-helix of LEMD2 functions uniquely in recruiting and activating a hybrid ESCRT protein named CHMP-7, as well as a unique role for Emr-1 in maintaining the NE permeability barrier. Their evidence further shows that CHMP-7 has an autonomous ‘backup’ role in preventing NE assembly failure. These results reveal separate roles for BAF binding to LEM-domain proteins, versus LEM2 binding to CHMPT7, during hole closure around MTs. Altogether these findings are a major step forward in understanding how spindle disassembly is coordinated with NE assembly and the multiple mechanisms used by cells to ensure the integrity of the NE barrier.

© 2023. Published by The Company of Biologists under the terms of the Creative Commons Attribution License (https://creativecommons.org/licenses/by/4.0/).

Comments for the author

The main issue for me was the condensed/terse writing in this manuscript, which often made it difficult to follow. E.g.:
Line 297-298: “indicates a unique function for LEM-2 from EMR-1 in germline development that is redundant to BAF-LEM binding.”
Lines 402-403: “Our data revealed an additional requirement for LEM-2-CHMP-7 when spindle MTs are present to stabilize the NE.”

Other comments:
Line 252-254: “The fact that LEM-2 and EMR-1 can localize to the NE independent of BAF-1 binding, albeit at significantly lower levels (Fig S3B, C), suggests these proteins can also function independent of BAF-1-binding at the NE.” Rephrase, since LEMD2 and Emr-1 have other known roles and partners at the NE, including lamins and chromatin-regulatory proteins.

As a major improvement toward reader understanding and appreciation of these findings, I urge the authors to provide a new ‘higher magnification’ molecular-level schematic that summarizes the binding interactions known so far for these proteins. This schematic could be loosely based on Figure S6D, but much simpler and more general, perhaps focused on how the wildtype proteins might interact and function during mitosis. E.g., show a few MTs in contact with chromatin; show LEMD2, which can bind both MTs and CHMP7; show CHMP7 recruiting the MT severing protein Spastin; show BAF located between chromatin and LEM-domain proteins at the NE; show CHMP7’s proposed independent role. The current schematics are appropriately detailed for readers to interpret these complicated fluorescence images, but are also confusingly un-general since they include the polar bodies, and both the oocyte-derived and sperm-derived nuclei, and the depictions of nuclear envelope membranes and chromatin are unclear.

First revision

Author response to reviewers' comments

We have uploaded a formatted PDF of the Response to Reviewers as Supplementary Information. Please refer to that document.

Response to Reviewers

We thank the reviewers for their insightful comments and constructive criticisms. We are happy that all reviewers found the manuscript to include important advances to the field and to be of interest to a broad audience of cell biologists.

In response to reviewers’ suggestions, we revised the text and figures to clarify our observations and improve readability. Edits in the manuscript text that pertain directly to the reviewers’ comments are marked in red.

Below, we address individual reviewers’ concerns:

Reviewer 1 Advance Summary and Potential Significance to Field:

This is a very well done and written manuscript on how the nuclear envelope seals after anaphase II of meiosis. It focuses nicely finding new mechanisms for how BAF, LEM proteins, and CHIMP7 function to form the new nuclear envelope in the presence of microtubule bundles. This is an important study that sheds new information on nuclear assembly. Interestingly, it used separation of function alleles in BAF-1 to identify slightly different roles for the LEM proteins EMR-1 and LEM-2, which were previously thought to function redundantly. It found a number of new conclusions: That BAF binding to LEM is not essential; That LEM-2 is more important than previously reported and that it plays a role independent of EMR-1. I particularly liked how it contrasted the roles of

© 2023. Published by The Company of Biologists under the terms of the Creative Commons Attribution License (https://creativecommons.org/licenses/by/4.0/).

these proteins in the female pronucleus which has a bundle of microtubules, and the male pronucleus which forms the NE at the same time in the absence of a MT bundle. Very cool. The figures are very well done and easy to follow. Given the maturity of the written manuscript and the figures, I suspect this has already been through the review process. I therefore have very few comments. It will be of interest to a broad group of cell biologists, including those interested in the nuclear envelope, micronuclei, DNA-membrane interactions, and early development including meiosis.

Reviewer 1 Comments for the Author:
No major revisions required!
Minor points:
1. I appreciate generation of the new lem-2 allele and setting the record straight on the tm1582 allele. Add allele names to the experiment. What is the new allele name? It should be near line 230. Also, add the allele name to figure 2g-h, is that the new allele? It is not clear. Also add the allele names to lines 354-355.

Response to Reviewer1 #1: We labeled the *lem-2* deletion allele (*sbw20*) in **new main Figures 2H, 2I, 4C, 4E, 5C, and 6A and supplementary Figures S2G-S2I**, **S4D-E, S6B, S6D and S6E**. We also added the allele name *lem-2*(*sbw20*) to the **main text pg. 9, line 228** where it is first mentioned **and throughout the rest of the manuscript.** We also added allele names in **new main text pg. 13, lines 364-378.**

2. In Figure 3B and S3G, the colors of the two lines are too close to each other. Please pick a color pair with more contrast.

Response to Reviewer1 #2: The line colors are now changed in **new Fig. 3B-E, S3B-C, and S3G.**

Reviewer 2 Advance Summary and Potential Significance to Field:

Barger and coworkers report that the interaction of DNA-binding protein Barrier-to-autointegration factor BAF with LEM domain proteins is required for efficient and rapid closure of nuclear membranes following segregation of chromosomes in meiosis II in C. elegans embryos. Previous investigations have demonstrated that the two C. elegans LEM domains EMR-1 (emerin) and LEM-2 (LEMD2) have both redundant and unique functions. This is also observed in this study. By performing high-resolution life microscopy on strains expressing endogenously tagged BAF-1, EMR-1, LEM-2 and CHMP-7, the authors demonstrate that all four proteins accumulate on the surface of the assembling oocyte-derived pronucleus at the site where final membrane closure takes place ("sealing plaque"). Depletion of BAF-1 by RNAi prevents accumulation of EMR-1 and LEM-2 and leads to the formation of highly aberrant pronuclei, which is in accordance with previous reports. Interestingly, a single point mutation in BAF-1 (L58R) that reduces the interaction with LEM domains has only a minor effect on fertility but leads to complete sterility in lem-2(0) mutants (but not when combined with emr-1(0)). The baf-1(L58R) mutation delays accumulation of EMR-1 and LEM-2 and the sealing plaque and, presumably in consequence, slower pronuclear envelope sealing and nuclear protein import. In the case of CHMP-7, accumulation in not delayed in baf-1(L58R) embryos but CHMP-7 distribution is irregular and pronuclear morphology is dramatically worsened in baf-1(L58R); chmp-7(RNAi) embryos. The effects on pronuclear morphology and pronuclear envelope sealing are stronger on the oocyte-derived pronucleus, which must seal a larger membrane gap after disassembly of meiotic microtubules compared to the sperm-derived pronucleus. Finally, the authors show that the WH domain of LEM-2 is require for efficient recruitment of CHMP-7 to reforming (pro-)nuclear envelopes, but not for nuclear accumulation of CHMP-7 nor viability.
The study is of high quality: the data are convincing, and the text provides a detailed description and discussion of the findings in the context of previous experiments performed in C. elegans and other organisms. I find that the manuscript provides an important advance in understanding the complex interplay between nuclear envelope proteins in nuclear assembly. However, I suggest the authors consider the following points prior to publication:

© 2023. Published by The Company of Biologists under the terms of the Creative Commons Attribution License (https://creativecommons.org/licenses/by/4.0/).

Reviewer 2 Comments for the Author:

1) The abstract argues that the relevance of BAF-LEM binding in membrane closure during mitosis is unknown and says in the following sentence that membrane closure is studied in meiosis in this study. This seems confusing. Is the mechanism the same in the two types of division?
Response to Reviewer2 #1: We edited the **new abstract on pg. 2 lines 13-25** to clarify this point. The abstract now reads:

> *"Barrier-to-autointegration (BAF) is a DNA binding protein that crosslinks chromatin to allow mitotic nuclear envelope (NE) assembly. The Lap2b-Emerin-Man1(LEM)-domain protein LEMD2 and ESCRTII/III hybrid protein CHMP7 close NE holes surrounding spindle microtubules (MTs). BAF binds LEM-domain family proteins, which repairs NE ruptures in interphase, but whether BAF-LEM binding participates in NE hole closure around spindle MTs is not known. Here, we take advantage of the stereotypical event of NE formation in fertilized C. elegans oocytes to show that BAF-LEM binding and LEM-2^{LEMD2}-CHMP-7 have distinct roles in NE closure around spindle MTs. LEM-2/EMR-1^{emerin} function redundantly with BAF-1 in NE closure. Compromising BAF- LEM binding revealed an additional role for EMR-1^{emerin} in maintenance of the NE permeability barrier. In the absence of BAF-LEM binding, LEM-2-CHMP-7 are required for NE assembly and embryo survival. The winged helix domain of LEM-2 recruits CHMP-7 to the NE in C. elegans and a LEM-2-independent nucleoplasmic pool of CHMP-7 also contributes to NE stability. Thus, NE hole closure surrounding spindle MTs requires redundant mechanisms that safeguard against failure in NE assembly to support embryogenesis."*

In the **new Introduction on pg. 4, lines 77-82,** we also include an explanation that NE sealing around meiotic spindle MTs is similar to mitosis, as pasted below:

> *"Here, we combine genetic analyses with live imaging of NE formation in C. elegans oocytes to determine the shared and unique functions for BAF, LEM-domain proteins and CHMP7 in NE sealing. Similar to mitotic NE formation, the NE seals around spindle MTs after anaphase of meiosis II in fertilized oocytes. This event establishes the oocyte-derived pronucleus and is the first instance of NE formation for the developing embryo. Using this system, we can also analyze formation of the sperm-derived pronucleus, which occurs at the same time but in the absence of MTs (Penfield et al., 2020; Fig. 1A)."*

2) All observations on protein localisation are based on mNG-tagged strains. By exclusively using endogenously tagged strains, expression levels are likely to reflect those of untagged proteins. Nevertheless, the mNG tag might still affect protein behaviour. Indeed, while both mNG-FLAG-BAF-1(wt) and BAF-1(L58R) are viable, the mNG-FLAG-BAF-1(L58R) strain is not viable in homozygosity, indicating an impact of mNG. Validating the accumulation at sealing plaques (or the absence thereof in baf-1 mutants) with antibodies against untagged proteins would strengthen the manuscript.

Response to Reviewer2 #2: We added a **new Fig. S2E** showing immunostaining of untagged BAF-1 and BAF-1(L58R) at the sealing plaque with a BAF-1 antibody that is described in the materials and methods. The instances in which we found oocytes at this stage were rare because of the transient nature of this event; however, in each case (n = 5), we found that the localization of BAF-1(L58R) to the sealing plaque was reduced compared to wild type, as described in the new **main text pg. 8, lines 195-199** that reads:

> *"In rare instances, we found oocytes at the stage of pronuclear formation by fixation and immunostaining. In each case, we detected lower levels of untagged BAF-1-L58R at the sealing plaque (Fig. S2E; n=5). However, immunostaining was not reliable in detecting BAF-1 fluorescence across fixed embryos, so we used live imaging of heterozygous mNG-tagged wild type and baf-1(L58R) strains to assess BAF-1 localization (Fig. S2E)."*

In response to *"the mNG tag might still affect protein behviour,"* please note that we used untagged strains for functional analysis (see **Figs. 2B, 2H and 2I and Figs. 3, 4, 5 and 6**). We only use the mNG tagged BAF-1 and BAF-1(L58R) to compare the localization of wild type BAF to the L58R mutant protein in **Figs. 2C-G**.

© 2023. Published by The Company of Biologists under the terms of the Creative Commons Attribution License (https://creativecommons.org/licenses/by/4.0/).

3) Related to the potential impact of mNG on fusion protein behaviour, why was mNG inserted at the N-terminus of EMR-1 and at the C-terminus of LEM-2? The ratio of NE/ER signal of LEM-2-mNG seems higher than that of mNG-EMR-1 – could the position of the tag influence this observation? Other studies in which EMR-1 was tagged at the C-terminus seem to have higher NE/ER signal ratio.

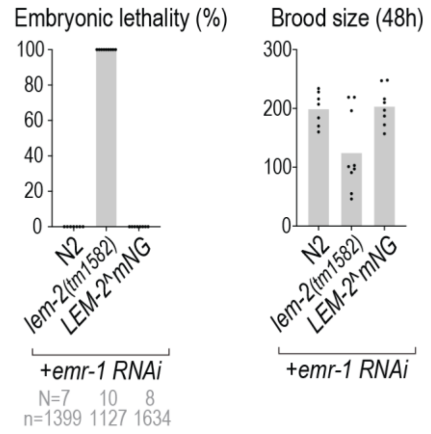

Response to Reviewer2 Figure: LEM-2^mNG is able to support embryonic viability in *emr-1(RNAi)* depleted embryos, which is not the case for the *lem-2(tm1582)*; *emr-(RNAi)* condition, as shown below.

Response to Reviewer2 #3a: mNG was inserted at the C-terminus of LEM-2 because it does not show any embryonic lethality or reduction in brood size with *emr- 1(RNAi)*-depletion, indicating that it is functional (**see Response to Reviewer2 Figure**).

We did not tag EMR-1 at its C-terminus because of its predicted topology as a tail-anchored protein (the C- terminal portion of the protein is predicted to insert into the membrane). To the best of our knowledge, we are the first to insert a fluorescent tag at the endogenous locus of *emr-1*. This may account for the differences in NE/ER ratio of EMR-1 that we observe compared to other studies that featured either bombardment strains with EMR-1 tagged at the N-terminus (PMID: 23444359 and PMID: 12475958) or single copy transgenic strains with EMR-1 tagged at the C-terminus (PMID: 25653391). In addition to differences in the genomic locus, signal to noise ratios, fluorescent protein tags used (mCherry and GFP versus mNG in our study), and/or tissues imaged may account for the different NE/ER ratios. We now cite these other studies and further clarify these differences in **main results pg. 6 lines 151- 154** that reads:

> *"The higher ER/NE ratio of mNG^EMR-1 that we observed (Fig. S1F), was not apparent in prior work using transgenic strains (Askjaer et al., 2002; Morales-Martínez et al., 2015; Pushpa et al., 2013). This difference may reflect the normal localization of EMR-1 from the endogenous locus or differences in the position or type of fluorescent protein fusions."*

Response to Reviewer2 #3b: *Note that in line 238 the order is reversed (EMR-1-mNG).*
Fixed (**new main text pg. 9, line 242**).

4a) In lines 203-204, the authors argue that binding to LEM domain proteins affect localisation of BAF-1 to the ER. However, the quantification of fluorescence signal seems to be based on ER + cytoplasm, i.e. not only ER.

Response to Reviewer2 #4a: Our interpretation that *"LEM-domain proteins affect localisation of BAF-1 to the ER"* is not based on the quantitation of the fluorescence signal, as the reviewer suggested, but on the qualitative observation that mNG^BAF-1 has a reticular localization in the cytoplasm, whereas mNG^BAF-1(L58R) does not (see **new Fig. 2C versus 2E**). To clarify this point, **pg. 8 in the main results lines 206-208** now reads:

© 2023. Published by The Company of Biologists under the terms of the Creative Commons Attribution License (https://creativecommons.org/licenses/by/4.0/).

> *"The reticular ER-localized signal also appeared reduced in mNG^ baf-1(L58R)/ baf-1 heterozygote embryos compared to mNG^baf-1 suggesting that BAF-1 may associate with the ER through binding to LEM domain proteins (Fig. 2C, E)."*

4b) Moreover, because mNG might stabilise BAF-1(wt) and/or destabilise BAF-1(L58R) the authors should demonstrate that mNG-FLAG-BAF-1 and mNG-FLAG-BAF-1(L58R) are expressed at the same level.

Response to Reviewer2 #4b: The reviewer is correct that the mNG tag may impact BAF-1 protein levels, as observed in the line scans showing that the fluorescent signal of the heterozygous mNG^FLAG-BAF-1(L58R) is uniformly lower than the heterozygous mNG-FLAG-BAF-1(WT) (see plots in **Figs. 2D versus E**).

We do not think that immunoblot analysis of the heterozygous mNG^FLAG-BAF-1(L58R) strain is necessary because of our fluorescence quantitation and text edits, as described below. In addition, this strain is not balanced making immunoblotting on heterozygous worms very difficult. As mentioned above in **Response to Reviewer2 #2**, all of our functional experiments are performed with the untagged *baf-1(L58R)* allele, which we show contains comparable levels of untagged BAF-1-L58R protein as wild type BAF-1 protein in whole worms (see **Fig. S2C** and **main text pg. 8, lines 201-202**).

We edited the text regarding this point in **new main text pg. 8, Line 199-202** that now reads:

> *"The mNG fluorescence levels were lower in heterozygote mNG^baf-1(L58R)/ baf-1 than in wild type mNG^baf-1/baf-1 embryos (Fig. 2C-E). Importantly, protein levels of untagged BAF-1-L58R in the baf-1(L58R) mutant strain that we use throughout this study are unchanged (Fig. S2C)."*

5) The discovery that the *lem-2(tm1582)* allele encodes a protein lacking aa 23-283 rather than just a short 53 aa peptide as previously assumed is quite important. However, a more detailed side-by- side comparison of phenotypes of the *tm1582* and the novel null allele should be provided.

Response to Reviewer2 #5: We added a figure panel showing that endogenous GFP^CHMP-7 localizes to the nuclear envelope in the *lem-2(tm1582)* mutant allele (**see new Fig. S6A and new text pg. 13, line 366-367**) for comparison with the *lem-2□□(sbw20)* strain (**see Fig, 6A**). These additional data provides sufficient background for our discovery that **"the *lem-2(tm1582)* allele encodes a protein lacking aa 23-283 rather than just a short 53 aa peptide as previously assumed"** and further underscores the importance of the WH domain in retaining CHMP-7 at the inner nuclear membrane in *C. elegans*.

6) Related to the previous point, why was the in-frame deletion allele *tm3468* of *lem-3* used rather than the stronger *mn155* allele?

Response to Reviewer2 #6: We chose the *lem-3(tm3468)* allele because it had been described to reduce the function of LEM-3 (PMID: 29463814). Our work is focused on BAF-mediated recruitment of transmembrane containing LEM-domain proteins during nuclear envelope sealing. LEM-3 is not a transmembrane containing LEM-domain protein and has not been implicated in nuclear envelope sealing, so we do not think it necessary to repeat this genetic experiment with the *lem-3(mn155)* mutant allele. In the text, we provide a clearer explanation of the in-frame *lem- 3* deletion allele (see **main text pg. 8, lines 224-227 and copied and pasted below**). We also changed the labels in **new Fig. 2H-I** to include the strain allele names for further clarity.

> *"The baf-1(L58R) mutant strain crossed to the emr-1 deletion or the lem-3(tm3468) mutant allele that is reduced in function resulted in only a slight decrease in brood size and embryonic viability (Fig. 2H, I) suggesting that these LEM proteins are not required for embryo and germline development when BAF-LEM binding is compromised."*

7) Some graphs include results of statistical tests while others do not (e.g. Fig S3, C vs G).

© 2023. Published by The Company of Biologists under the terms of the Creative Commons Attribution License (https://creativecommons.org/licenses/by/4.0/).

Response to Reviewer2 #7: We now included statistical analyses in the **main figure legends of Figs. 3C-E, Fig. 4C, Fig. 5C, Fig. 6E, F** and **supplementary figure legends Fig. S1A, S2D, S3G, S4C, S5F, S6E.**

8) Are the differences in nuclear import of GFP-NLS-LacI in Figure 4C and S4C statistically significant?

Response to Reviewer2 #8: Yes, the difference in nuclear import of GFP-NLS between the control and *baf-1* mutant is statistically significant in the oocyte-derived pronucleus (**see Fig. 4C and main figure legend for Fig. 4C**) and not significant in the sperm-derived pronucleus (**see Fig. S4C and main figure legend for Fig. S4C**). We now include statistical significance for these data with details added in the figure legends pertaining to these figure panels.

9) Regarding the argument in lines 282-284: "Sterility resulting from loss of lem-2 in baf-1(L58R) worms prevented us from genetically testing the consequences resulting from loss of lem-2 in baf-1(L58R) embryos." This could be investigated by RNAi against lem-2 as for emr-1 and lem-4 in Fig 4E.

Response to Reviewer2 #9: This is a great suggestion and we have now conducted this experiment. The additional data describing nuclear envelope sealing assessed by exclusion of GFP-tubulin in the oocyte- and sperm-derived pronucleus with the *lem-2(sbw20)* allele and RNAi-depletion of *lem-2* in the *baf-1(L58R)* mutant is reported in **new Fig. 4E** with corresponding images of the same condition with an ER marker in **new Fig. S4D.** Also see new text **pg. 10-11, lines 288-306.**

Reviewer 3 Advance Summary and Potential Significance to Field:
This is an extremely careful and rigorous cell biological and genetic analysis of a challenging stage in nuclear envelope (NE) assembly, using the best possible biological system (meiotic prophase-II in C. elegans embryos). This manuscript reports the mechanisms of NE membrane sealing in the 'core' region where spindle microtubules (MTs) create large holes that interfere with the enclosure of chromosomes by nuclear membranes, focusing on the intertwined roles of BAF-1 and two 'redundant' LEM-domain proteins (Emr-1/emerin and LEMD2), as well as Lem3. They made careful use of a BAF-1 mutation (L58R) defective in binding to LEM-domain proteins. The authors reveal a long-standing annotation mistake in the field about the null phenotype of LEMD2, which allowed them to discover that the winged-helix of LEMD2 functions uniquely in recruiting and activating a hybrid ESCRT protein named CHMP-7, as well as a unique role for Emr-1 in maintaining the NE permeability barrier. Their evidence further shows that CHMP-7 has an autonomous 'backup' role in preventing NE assembly failure. These results reveal separate roles for BAF binding to LEM-domain proteins, versus LEM2 binding to CHMPT7, during hole closure around MTs. Altogether these findings are a major step forward in understanding how spindle disassembly is coordinated with NE assembly and the multiple mechanisms used by cells to ensure the integrity of the NE barrier.

Reviewer 3 Comments for the Author:
(1) The main issue for me was the condensed/terse writing in this manuscript, which often made it difficult to follow. E.g.:

Response to Reviewer3 #1a: Thank you for your suggestion and we appreciate the selected examples. We made several edits to the language/sentence structure as highlighted in red throughout the manuscript to make the text easier to follow.

Regarding the specific examples:

Line 297-298: "indicates a unique function for LEM-2 from EMR-1 in germline development that is redundant to BAF-LEM binding."

Response to Reviewer3 #1b: We removed this sentence as it was repetitive with other portions of the main text (**see pg. 9, lines 235-237**).

Lines 402-403: "Our data revealed an additional requirement for LEM-2-CHMP-7 when spindle MTs

© 2023. Published by The Company of Biologists under the terms of the Creative Commons Attribution License (https://creativecommons.org/licenses/by/4.0/).

are present to stabilize the NE."

Response to Reviewer3 #1c: We removed the sentence in lines 402-403 to improve readability in the **new Discussion pg. 14-16**.

Other comments:
(2) Line 252-254: "The fact that LEM-2 and EMR-1 can localize to the NE independent of BAF- 1 binding, albeit at significantly lower levels (Fig S3B, C), suggests these proteins can also function independent of BAF-1-binding at the NE." Rephrase, since LEMD2 and Emr-1 have other known roles and partners at the NE, including lamins and chromatin-regulatory proteins.
Response to Reviewer3 #2: We agree. We revised this sentence and added appropriate citations (see **new main text pg. 9 lines 257-259**) that now reads:

> *"consistent with reports that these proteins have binding partners at the NE other than BAF-1, including lamins and chromatin-regulatory proteins (Caravia et al., 2022; Holaska and Wilson, 2007; Moser et al., 2020)."*

(3) As a major improvement toward reader understanding and appreciation of these findings, I urge the authors to provide a new 'higher magnification' molecular-level schematic that summarizes the binding interactions known so far for these proteins. This schematic could be loosely based on Figure S6D, but much simpler and more general, perhaps focused on how the wildtype proteins might interact and function during mitosis. E.g., show a few MTs in contact with chromatin; show LEMD2, which can bind both MTs and CHMP7; show CHMP7 recruiting the MT severing protein Spastin; show BAF located between chromatin and LEM-domain proteins at the NE; show CHMP7's proposed independent role. The current schematics are appropriately detailed for readers to interpret these complicated fluorescence images, but are also confusingly un-general since they include the polar bodies, and both the oocyte-derived and sperm-derived nuclei, and the depictions of nuclear envelope membranes and chromatin are unclear.

Response to Reviewer3 #3: We appreciate this comment and have now significantly revised our model to be easier to interpret (see **new Fig. S6F**). We include MTs in contact with chromatin and show BAF located between chromatin and LEM-domain proteins. We also depict the membrane closure of the NE hole in 3D. Overall, we believe the depictions of the NE, chromatin, and protein placement are clearer (inset panels on the left).

We chose to include the details involving the oocyte- and sperm-derived pronuclei because it highlights our unique model of *C. elegans* meiosis in which two pronuclei share a cytoplasm, yet only the oocyte-derived pronucleus undergoes a major sealing event around spindle MTs. To assist readers, we provide a more general schematic of the embryo phenotypes with short descriptions below (see **new Fig. S6F, right**). We prefer not to include Spastin because the ESCRT-III-dependent recruitment of Spastin to the reforming NE has not been reported in *C. elegans*. Furthermore, katanin is considered the primary MT severing protein at this time point (PMID: 32412594).

<u>Second decision letter</u>

MS ID#: JOCES/2023/261385

MS TITLE: Nuclear envelope assembly relies on CHMP-7 in the absence of BAF-LEM-mediated hole closure

AUTHORS: Sarah R Barger, Lauren Penfield, and Shirin Bahmanyar
ARTICLE TYPE: Research Article

I am happy to tell you that your manuscript has been accepted for publication in Journal of Cell Science, pending standard ethics checks.

© 2023. Published by The Company of Biologists under the terms of the Creative Commons Attribution License (https://creativecommons.org/licenses/by/4.0/).
